# Role of mast cell-derived exosomes in exacerbating neuronal injury of experimental cerebral malaria

**DOI:** 10.1186/s13071-025-06863-3

**Published:** 2025-07-01

**Authors:** Qianru Wang, Xiumei Mo, Hua Li, Mingqiu Ye, Guojun Fei, Pinru Chen, Yongfei Wang, Xinpeng Hou, Jiajing He, Wenbin Liu, Jie Wang, Hui Yin, Zujun Deng, Xiaobao Jin, Zhenlong Liu, Qi Wang, Bo Huang

**Affiliations:** 1 Guangdong Provincial Key Laboratory of Pharmaceutical Bioactive Substances, Guangdong Pharmaceutical University, Guangzhou, 510006 People’s Republic of China; 2 Department of Critical Care Medicine, Shenzhen Bao’an District Songgang People’s Hospital, Shenzhen, 518105 China; 3 Department of Critical Care Medicine, Foshan Sanshui District People’s Hospital, Foshan, 528199 China; 4 Guangzhou Chest Hospital, Guangzhou, 510095 People’s Republic of China; 5 School of Life Science and Technology, Jinan University, Guangzhou, 510632 People’s Republic of China; 6 Laboratory Animal Center, Guangdong Pharmaceutical University, Guangzhou, 510006 People’s Republic of China; 7 Division of Experimental Medicine, Department of Medicine, McGill University, Montreal, QC Canada; 8 School of Basic Medical Science, Guangdong Pharmaceutical University, Guangzhou, 510006 People’s Republic of China

**Keywords:** Cerebral malaria, Mast cells, Exosomes, Neuron, Endoplasmic reticulum stress

## Abstract

**Background:**

Cerebral malaria (CM), a fatal neurological complication of *Plasmodium falciparum* infection, is partially driven by neuronal injury. Emerging evidence highlights exosomes as vital mediators of mast cell–neuron interactions in neurological disease progression. While mast cells and their exosomes were previously shown to exacerbate experimental cerebral malaria (ECM) severity, the specific role of mast cell-derived exosomes in CM-associated neuronal injury remains unclear.

**Methods:**

Exosomes were isolated from resting and lipopolysaccharide (LPS)-activated P815 mast cells (denoted as *RE* and *AE*, respectively) and characterized. These exosomes were administered to ECM mice and *Plasmodium berghei* ANKA (*Pb*A)-infected red blood cell (iRBC)-stimulated neuronal HT-22 cells to investigate their functional impact and mechanisms.

**Results:**

Both *RE* and *AE* exhibited spherical morphology (20–100 nm diameter) and expressed exosomal markers (CD9, CD63, and CD81). Compared to infected controls, *RE* and *AE* treatments significantly reduced survival time, increased ECM incidence, and exacerbated brain pathology, blood–brain barrier disruption, neuronal injury, and apoptosis. Furthermore, *RE* and *AE* administration elevated messenger RNA (mRNA) levels of pro-inflammatory cytokines (interleukin [IL]-6, tumor necrosis factor alpha [TNF-α], and IL-1β) and increased numbers of neurons expressing endoplasmic reticulum (ER) stress markers (GRP78, CHOP, p-IRE1, XBP-1). Notably, *AE* treatment induced higher morbidity/mortality rates, more severe neuronal injury, and greater ER stress marker expression than *RE*. In vitro, *RE*-treated iRBC-stimulated neuronal HT-22 cells showed higher GRP78, CHOP, and XBP-1 mRNA levels than *AE*-treated cells. MicroRNA (miRNA) sequencing revealed three downregulated miRNAs (miR-330-3p, miR-185-5p, and miR-379-5p) and six upregulated miRNAs (miR-155-5p, miR-423-3p, miR-187-3p, miR-29c-3p, miR-188-5p, miR-192-5p) in *AE* versus *RE*, all previously implicated in targeting GRP78, CHOP, or XBP-1.

**Conclusions:**

Mast cell-derived exosomes, particularly those from activated cells (*AE*), exacerbated ECM neuronal injury through partial activation of ER stress pathways.

**Graphical Abstract:**

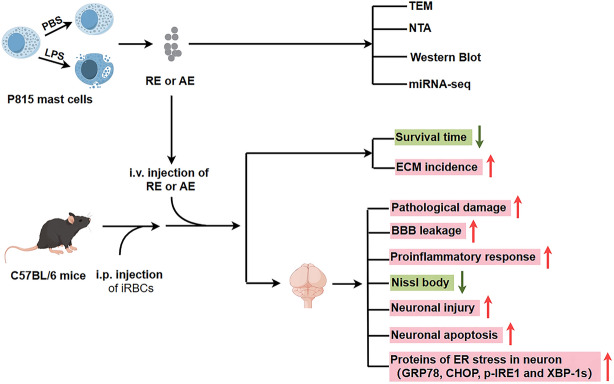

**Supplementary Information:**

The online version contains supplementary material available at 10.1186/s13071-025-06863-3.

## Background

Malaria, a mosquito-borne parasitic disease caused by protozoans of the genus *Plasmodium*, disproportionately affects low-income countries in sub-Saharan Africa, with an estimated 249 million cases and 608,000 deaths reported globally in 2022 [1]. Cerebral malaria (CM), a rapidly progressive neurological complication of *Plasmodium falciparum* infection, carries a mortality rate approaching 100% if untreated, while even with artesunate therapy (the gold-standard antimalarial), case fatality remains at 20–30%. Notably, approximately 25% of survivors develop long-term neurocognitive sequelae [2]. Epidemiological data indicated that CM accounts for around 2% of *P. falciparum* malaria cases yet contributes to over 90% of malaria-related deaths [2]. Pathologically, CM is characterized by cerebral microvascular sequestration of infected red blood cells (iRBCs), which trigger impaired cerebral perfusion, blood–brain barrier (BBB) disruption, neuronal injury, and dysregulated neuroinflammation [3, 4]. Emerging evidence positions neuronal injury not only as a central driver of acute CM pathogenesis but also as a determinant of long-term neurological deficits in survivors [5, 6]. While studies have implicated programmed cell death pathways (e.g., apoptosis and ferroptosis) in CM-associated neuronal injury [7, 8], the precise molecular mechanisms remain poorly defined, hindering targeted therapeutic development.

Mast cells, innate immune cells characterized by basophilic granules and limited phagocytic capacity, are ubiquitously distributed near capillaries and nerve terminals across tissues [9]. Beyond their canonical role in immunoglobulin E (IgE)-mediated allergies, mast cells orchestrate innate and adaptive immune responses during parasitic infections via secretion of diverse mediators (e.g., cytokines) [9]. Intriguingly, research has shown that antimalarial agents (artesunate, ketotifen, and mefloquine) not only exhibit direct parasiticidal effects but also suppress mast cell degranulation, migration, and adhesion, suggesting mast cell involvement in host–parasite interplay [10–13]. Clinical and experimental studies have revealed mast cell hyperplasia and degranulation in the skin, intestine, lymph nodes, and brain of malaria patients and murine models, correlating with increased parasitemia and disease severity [14, 15]. In *Plasmodium berghei* ANKA (*Pb*A)-infected mice, pharmacological activation of mast cells (compound 48/80) accelerated mortality, increased parasite load, and exacerbated tissue damage, whereas mast cell stabilization (disodium cromoglycate) ameliorated these outcomes [15]. Mechanistically, mast cell-derived Flt3 and histamine worsened parasite burden, tissue pathology, intestinal permeability, and BBB integrity in *Pb*A-infected mice [16, 17], whereas chymase, protease 4, and interleukin (IL)−10 from mast cells paradoxically enhanced intestinal barrier function [18, 19]. Despite sparse distribution in brain regions (e.g., area postrema, meninges, choroid plexus, and thalamic–hypothalamic axis), cerebral mast cells were found to critically regulate neurodevelopment, motivation, and cognition under homeostasis through neurovascular cross-talk [20]. Conversely, mast cell dysfunction was implicated in neuropathologies such as cerebral ischemia, hemorrhage, epilepsy, and stroke [20–22]. Emerging evidence has implicated exosomes—nanoscale extracellular vesicles—as key mediators of mast cell–neuron communication, driving neuropathological cascades [23, 24]. Our prior work established that exosomes from P815 mast cells exacerbated experimental cerebral malaria (ECM) severity by promoting brain microvascular endothelial activation and BBB breakdown [25]. However, the mechanisms by which mast cell-derived exosomes amplify neuronal injury in ECM remain undefined.

Endoplasmic reticulum (ER), a pivotal intracellular organelle for lipid biosynthesis and protein quality control, regulates cellular homeostasis through its unfolded protein response (UPR) [26]. Accumulation of misfolded/unfolded proteins triggers UPR activation, inducing a state referred to as ER stress. Notably, ER stress is shown to exert dual effects: transient activation resolved protein misfolding and promoted cell survival, whereas chronic ER stress drove pathological cell dysfunction [26]. Emerging evidence has implicated ER stress in neuronal injury across neurodegenerative disorders [27–29]. In an ECM model, upregulated GRP78 (a canonical ER stress marker) was detected in brain tissues, suggesting that ER stress exacerbates neuronal injury in ECM [30]. Mechanistically, mast cells induced ER stress and UPR activation in colorectal cancer cells via cystatin C secretion [31], and similarly drove ER stress-mediated endothelial dysfunction and BBB disruption in sickle cell disease models [32]. Critically, exosomes from diverse cell types (including mast cells) are known to modulate ER stress pathways [33, 34], prompting the hypothesis that mast cell-derived exosomes may amplify ECM neuronal injury through ER stress activation.

To test this, we isolated exosomes from resting and lipopolysaccharide (LPS)-activated P815 mast cells (designated as *RE* and *AE*, respectively) and characterized their properties. Using a murine ECM model and *Pb*A-iRBC-stimulated HT-22 neuronal cells, we evaluated the functional impact of *RE*/*AE* on neuronal injury and ER stress pathways. Our results demonstrated that both *RE* and *AE* exacerbated ECM-associated neuronal injury, partially via ER stress activation. These findings establish mast cell-derived exosomes as novel mediators of neuropathology in CM, highlighting their therapeutic potential as targets for intervention.

## Methods

### Animals and parasites

Female C57BL/6 mice (aged 6–8 weeks) were obtained from the Guangdong Experimental Animal Center and housed under specific-pathogen-free (SPF) conditions in the Animal Center of Guangdong Pharmaceutical University, with controlled temperature (22 ± 1 °C), humidity (50–60%), and a 12-h light/dark cycle. *Plasmodium berghei* ANKA-infected red blood cells (*Pb*A-iRBCs) were generously provided by Prof. Jianping Song (Guangzhou University of Chinese Medicine, China) and stored at −80 °C. Cryopreserved *Pb*A-iRBCs were rapidly thawed at 37 °C and intraperitoneally (i.p.) administered to 6-week-old female C57BL/6 donor mice (200 µl/mouse, 1 × 10^6^ iRBCs) to maintain parasite virulence and genetic stability. Parasitemia progression was monitored daily via Giemsa-stained thin blood smears. When peripheral iRBC infection rates reached ~10% in the donor mice, the harvested iRBCs (1 × 10^6^ in 200 µl phosphate-buffered saline [PBS]) were subsequently i.p. transferred to Naïve recipient mice for parasite expansion. All experimental protocols complied with the National Guidelines for the Care and Use of Laboratory Animals and were approved by the Animal Ethics Committee of Guangdong Pharmaceutical University (Approval No. gdpulac2023001).

### Isolation and characterization of mast cell-derived exosomes

The murine mastocytoma cell line P815 was procured from the Stem Cell Bank of the Chinese Academy of Sciences (Cat. No. SCSP-516) and maintained in RPMI-1640 complete medium (Cat. No. 11875093, Gibco) supplemented with 10% exosome-depleted fetal bovine serum (FBS; Cat. No. 2720801, Gibco) and 1% penicillin–streptomycin (Cat. No. 15140148, Gibco) at 37 °C under 5% CO_2_. To generate activated exosomes, P815 cells (~80% confluence) were stimulated with 1 μg/ml LPS in PBS for 24 h [35, 36], while resting controls received PBS alone. Conditioned media from LPS-activated or resting P815 cells (designated as *AE* and *RE*, respectively) were collected and mixed with Total Exosome Isolation Reagent (Cat. No. 4478359, Invitrogen) at a 2:1 (*v*/*v*) ratio, vortexed, and incubated overnight at 4 °C. Exosome pellets were obtained by centrifugation at 10,000×*g* for 1 h at 4 °C, resuspended in 100 μl PBS, and stored at −80 °C.

For exosome characterization, negative-staining transmission electron microscopy (TEM) was performed as described previously [37]. Briefly, exosome suspensions were adsorbed onto copper grids, fixed with 2% phosphotungstic acid for 1 min, and imaged using an FEI Tecnai G2 Spirit Twin microscope (Thermo Fisher Scientific, USA). Nanoparticle tracking analysis (NTA) was conducted on a NanoSight LM10 system (Malvern Panalytical, UK) to determine size distribution. Western blotting confirmed exosomal markers (CD9, CD63, and CD81) using the following protocol: Exosomal proteins were lysed in RIPA buffer, quantified via BCA assay (Beyotime, China), separated on 10% sodium dodecyl sulfate–polyacrylamide gel electrophoresis (SDS-PAGE) gels, and transferred to polyvinylidene fluoride (PVDF) membranes. After blocking with 5% skim milk, membranes were incubated overnight at 4 °C with primary antibodies (CD9: Cat. No. 205971-AP, CD63: Cat. No. 256821-AP, CD81: Cat. No. 278551-AP; β-actin [as the loading control]: Cat. No. 60008-1-Ig; Proteintech, China) followed by horseradish peroxidase (HRP)-conjugated goat anti-rabbit IgG (Cat. No. S0001, Affinity) for 1 h. Signals were detected using a ChemiDoc MP Imaging System (Bio-Rad, USA).

### Exosomal miRNA sequencing analysis

MicroRNA (miRNA) profiles of exosomes between *AE* and *RE* were analyzed by high-throughput sequencing on an Illumina HiSeq 2500 platform (Illumina, Inc., San Diego, CA, USA). Total RNA was extracted from exosomes using TRIzol reagent (Cat. No. 9109, TaKaRa Bio) and assessed for quality using a NanoDrop ND-1000 spectrophotometer (Thermo Fisher Scientific, USA) with A260/A280 ratios of 1.8–2.0, followed by RNA integrity verification using an Agilent 2100 Bioanalyzer (Agilent Technologies, USA). Small RNA libraries were prepared by ligating 3′ and 5′ adapters to RNA fragments, reverse-transcribing into complementary DNA (cDNA), and amplifying via polymerase chain reaction (PCR). Amplified products (135–155 base pairs [bp]) were size-selected via PAGE purification, and libraries were sequenced on an Illumina NextSeq 500 platform (Illumina, USA) with single-end 75-bp reads. Reads of 18–30 nucleotides were retained for downstream analysis. Differentially expressed miRNAs of exosomes between *AE* and *RE* were identified using R 3.5.3 (R Foundation) with criteria of |log2(fold change)|> 1 and *P* < 0.05. A clustered heatmap was generated to visualize miRNA expression patterns*.*

### Experimental design and exosome administration

A total of 117 C57BL/6 mice were randomly allocated into six groups (Naive, *RE*, *AE*, *Pb*, *Pb*+*RE*, *Pb*+*AE*). The experimental timeline was adapted from previous studies [25, 37, 38] with protocol optimizations. In three independent and separate experimental cohorts (*n* = 39 mice per cohort), Naïve (*n* = 4), *RE* (*n* = 4), and *AE* (*n* = 4) groups received daily intravenous (i.v.) injections of 50 μl PBS, 100 μg *RE*, or 100 μg *AE* (resuspended in PBS), respectively, without *Pb*A infection. *Pb* (*n* = 9), *Pb*+*RE* (*n* = 9), and *Pb*+*AE* (*n* = 9) groups were injected i.p. with 1 × 10^6^ *Pb*A-iRBCs followed by daily i.v. administration of 50 μl PBS, 100 μg *RE*, or 100 μg *AE*, respectively. Parasitemia was quantified daily from day 3 post-infection (p.i.) via Giemsa-stained thin blood smears (tail vein blood), calculated as (iRBCs/total RBCs) × 100%. ECM was defined by neurological symptoms (hemiparesis, ataxia, absent reflexes, and coma) accompanied by mortality within 24 h of symptom onset (6–11 days p.i.), whereas mice that evade neurological symptoms may die later (12–20 days p.i.) due to hyperparasitemia-driven systemic pathology, including anemia and multi-organ failure. Survival rates, parasitemia progression, and ECM incidence were recorded for all infected groups. ECM mice meeting clinical criteria were randomly selected for subsequent analyses.

### Hematoxylin and eosin (H&E), Nissl, or Fluoro-Jade B (FJB) Staining

To evaluate the pathological effects of *RE* and *AE* in ECM, brain tissues from six ECM mice per group (*n* = 2 mice per experimental replicate; three independent replicates) were fixed in 4% neutral-buffered formalin for 48 h, processed through graded ethanol-xylene dehydration, embedded in paraffin, and sectioned into 4-μm-thick non-consecutive coronal slices. Following deparaffinization in xylene and rehydration through an ethanol gradient, sections were subjected to hematoxylin (Cat. No. 517-28-2, Sigma-Aldrich) and eosin (Cat. No. 17372-87-1, Sigma-Aldrich) for 5 min each, or Nissl dye (Cat. No. 10510-54-0, Sigma-Aldrich) for 10 min at 25 °C, and imaged using a Leica DM2500B microscope at ×400 magnification. For FJB staining, the sections were incubated with FJB working solution (Cat. No. AG310, Merck) at 4 °C, followed by 10-min DAPI counterstaining. FJB-stained sections were mounted with anti-fade polyvinylpyrrolidone medium (Cat. No. P0126, Beyotime) and visualized under a Zeiss LSM780 confocal microscope with identical magnification. Quantification of pyknotic nuclei (H&E), Nissl^+^ neurons, and FJB^+^ degenerating neurons was performed by two blinded investigators across 20 randomly selected fields per section (*n* = 6 ECM mice/group).

### Evans blue permeability assay

To evaluate the effects of *RE* and *AE* on BBB integrity in ECM mice, we conducted Evans blue (EB) extravasation measurements using an established protocol with minor modifications [25]. When *Pb*A-infected mice developed neurological symptoms (ECM criteria), six ECM animals per group (*n* = 2 mice per experimental replicate; three independent replicates) received i.v. injection of 0.2 ml 0.5% EB dye solution (Cat. No. E8010, Solarbio) through the tail vein. Following 60 min circulation, mice were anesthetized with isoflurane inhalation and underwent transcardial perfusion with 10 ml heparinized saline (10 U/ml) to remove intravascular dye. Brains were immediately harvested, weighed, and photographed for macroscopic evaluation. Tissue samples were then homogenized in 20 ml formamide and incubated at 37 °C for 48 h to extract extravasated dye. EB dye concentration in formamide supernatants was quantified by absorbance measurement at 620 nm using a microplate reader (iMark, Bio-Rad). Dye content was normalized to tissue mass using a standard curve and expressed as nanograms EB per milligram brain tissue (ng/mg).

### Quantitative real-time PCR analysis of neuroinflammatory markers

To assess the effects of *RE* and *AE* on neuroinflammation in ECM mice, we quantified messenger RNA (mRNA) levels of pro-inflammatory cytokines (IL-6, tumor necrosis factor alpha [TNF-α], and IL-1β) in the cerebral cortex and thalamus using quantitative real-time PCR (qPCR). Cerebral cortex and thalamus samples were collected from uninfected mice in the Naive, *RE*, and *AE* groups, and ECM in the *Pb*, *Pb*+*RE*, and *Pb*+*AE* groups (*n* = 6 mice/group). Total RNA was isolated using TRIzol reagent (Cat. No. 9109, TaKaRa Bio) and quantified using a NanoDrop 2000 spectrophotometer. Reverse transcription was performed with the PrimeScript™ 1 st Strand cDNA Synthesis Kit (Cat. No. AG11711, Accurate Biology) using 1 μg total RNA per reaction. The reaction system contained 10 µl of SYBR Green pro Taq HS Premix (Cat. No. AG11701, Accurate biology), 2 μl of cDNA (100 ng/μl), 0.5 μl of each primer, and 7 µl of dH_2_O. qPCR amplification was performed on a CFX96 Real-Time System (Bio-Rad, USA) under the following conditions: 95 °C for 30 s (initial denaturation), followed by 40 cycles of 95 °C for 5 s and 60 °C for 30 s. Primer sequences were adapted from previous work [37]: IL-6 (F: 5′-CTTCTTGGGACTGATGCTGGTGAC-3′, R: 5′-TCTGTTGGGAGTGGTATCCTCTGTG-3′), TNF-α (F: 5′-CGCTCTTCTGTCTACTGAACTTCGG-3′, R: 5′-GTGGTTTGTGAGTGTGAGGGTCTG-3′), IL-1β (F: 5′-CACTACAGGCTCCGAGATGAACAAC-3′, R: 5′-TGTCGTTGCTTGGTTCTCCTTGTAC-3′), and β-actin (F: 5′-TATGCTCTCCCTCACGCCATCC-3′, R: 5′-GTCACGCACGATTTCCCTCTCAG-3′). The mRNA levels of pro-inflammatory cytokines (IL-6, TNF-α, and IL-1β) were calculated using the 2^−ΔΔCt^ method with β-actin as the endogenous reference gene.

### TUNEL^+^-NeuN^+^ co-staining for neuronal apoptosis assessment

To evaluate the effects of *RE* and *AE* on neuronal apoptosis in ECM mice, TUNEL (Terminal deoxynucleotidyl transferase dUTP Nick End Labeling)-NeuN co-staining was performed on brain sections across experimental groups (*n* = 6 mice/group). Deparaffinized and rehydrated sections underwent antigen retrieval via microwave heating in sodium citrate buffer (pH 6.0), followed by endogenous peroxidase blocking with 3% H_2_O_2_ and nonspecific site blocking with 10% normal goat serum (Cat. No. AR0009, Boster Biological Technology). Sections were incubated with rabbit anti-NeuN polyclonal antibody (1:300; Cat. No. GB11138, Servicebio) at 4 °C overnight, then with Alexa Fluor 594-conjugated goat anti-rabbit IgG (1:300; Cat. No. 8889, Cell Signaling Technology) at 37 °C for 1 h. After PBS washes, apoptotic cells were labeled using the TUNEL detection kit (Cat. No. G1501, Servicebio), and nuclei were counterstained with DAPI (2 μg/ml). Imaging was conducted using a Zeiss LSM780 confocal microscope at ×400 magnification: TUNEL^+^ apoptotic cells emitted green fluorescence, NeuN^+^ neurons displayed red fluorescence, and dual-positive apoptotic neurons exhibited merged yellow signals. Two blinded investigators quantified TUNEL^+^-NeuN^+^ cells in approximately 20 randomly selected fields per section, with results presented as apoptotic neurons per field.

### Dual immunofluorescence staining

To evaluate the effects of *RE* and *AE* on neuronal ER stress in ECM mice, dual immunofluorescence staining was performed to detect NeuN^+^-GRP78^+^, NeuN^+^-p-IRE1^+^, NeuN^+^-XBP-1s^+^, and NeuN^+^-CHOP^+^ co-labeled neurons across experimental groups (*n* = 6 mice/group). Deparaffinized and rehydrated brain sections underwent antigen retrieval in sodium citrate buffer at 95 °C for 10 min, followed by endogenous peroxidase inactivation with 3% H_2_O_2_ for 25 min and blocking with 10% goat serum for 30 min. Sections were sequentially incubated with primary antibodies: monoclonal mouse anti-NeuN (1:300; Cat. No. GB15138, Servicebio) at 4 °C overnight, then HRP-conjugated goat anti-mouse IgG (1:200; Cat. No. 8125P, Cell Signaling Technology) at 37 °C for 30 min. After PBS washes, sections were incubated overnight at 4 °C with species-specific primary antibodies against ER stress markers: mouse anti-GRP78 (1:500; Cat. No. GB12098, Servicebio), rabbit anti-p-IRE1 (1:300; Cat. No. ab48187, Abcam), rabbit anti-XBP-1s (1:400; Cat. No. 40435S, Cell Signaling Technology), or mouse anti-CHOP (1:400; Cat. No. 2895, Cell Signaling Technology). Alexa Fluor 488-conjugated secondary antibodies (goat anti-rabbit or anti-mouse IgG; Cell Signaling Technology) were applied at 37 °C for 60 min in the dark, followed by DAPI counterstaining (2 μg/ml). Sections were mounted with anti-fade medium and imaged using a Zeiss LSM780 confocal microscope at ×400 magnification. NeuN^+^ neurons emitted green fluorescence, ER stress markers showed red fluorescence, and co-localized signals appeared yellow. Two blinded investigators quantified double-positive cells in approximately 20 randomly selected fields per section, with results expressed as co-labeled cells per field.

### In vitro co-culture of iRBC-stimulated neuronal HT-22 cells with *RE* or *AE* treatment

HT-22 neuronal cells (Cat. No. GNM47, Stem Cell Bank, Chinese Academy of Sciences) were cultured at 37 °C with 5% CO_2_ in Dulbecco’s modified Eagle medium (DMEM; Cat. No. 10566016, Gibco) supplemented with 10% exosome-depleted FBS (Cat. No. 2720801, Gibco) and 100 U/ml penicillin–streptomycin (Cat. No. 15140148, Gibco). To evaluate ER stress-related gene expression (GRP78, CHOP, and XBP-1s) in iRBC-stimulated HT-22 cells under *RE* or *AE* treatment, cells were seeded in six-well plates at 2 × 10^5^ cells/ml and stimulated with 5.0 × 10^6^ iRBCs/well. Treatments with 100 μl ice-cold PBS containing *RE* (100 μg/ml), *AE* (100 μg/ml), or vehicle control were applied for 24 h or 48 h. Cell pellets were collected after PBS washing, followed by total RNA extraction using TRIzol reagent (Cat. No. 9109, TaKaRa Bio) and reverse transcription with the PrimeScript™ 1 st Strand cDNA Synthesis Kit (Cat. No. AG11711, Accurate Biology). qPCR was performed using SYBR Green Master Mix (Cat. No. 6110B, TaKaRa Bio) on a CFX96 Real-Time System (Bio-Rad, USA), with β-actin as the endogenous control. Primer sequences were as follows: GRP78 (F: 5′-CTTCTCAGCATCAAGCAAGGATTG-3′, R: 5′-TCATGGTAGAGCGGAACAGGTC-3′), CHOP (F: 5′-TGAAGGAGAAGGAGC AGGAGAAC-3′, R: 5′-GAGCCCGCCGTGTGGTC-3′), XBP-1s (F: 5′-GGGAGTGGAGTAAGGCTGGTG-3′, R: 5′-AGAGGCAACAGTGTCAGAGTCC-3′), and β-actin (F: 5′-TATGCTCTCCCTCACGCCATCC-3′, R: 5′-GTCACGCACGATTTCCCTCTCAG-3′). Data were analyzed via the 2^−ΔΔCT^ method. Four independent biological replicates were conducted.

### Statistical analysis

Statistical analyses were performed using GraphPad Prism v5.0 (GraphPad Software). Data are expressed as mean ± standard deviation (SD). Differences between two groups were analyzed using unpaired Student’s *t*-tests, while the one-way ANOVA test was applied for comparisons involving three or more groups. Parasitemia dynamics were evaluated via a time series analysis test. Survival curves were compared using the log-rank (Mantel–Cox) test. A *P*-value < 0.05 was considered statistically significant.

## Results

### Characterizations of *RE* and *AE*

**Fig. 1:**
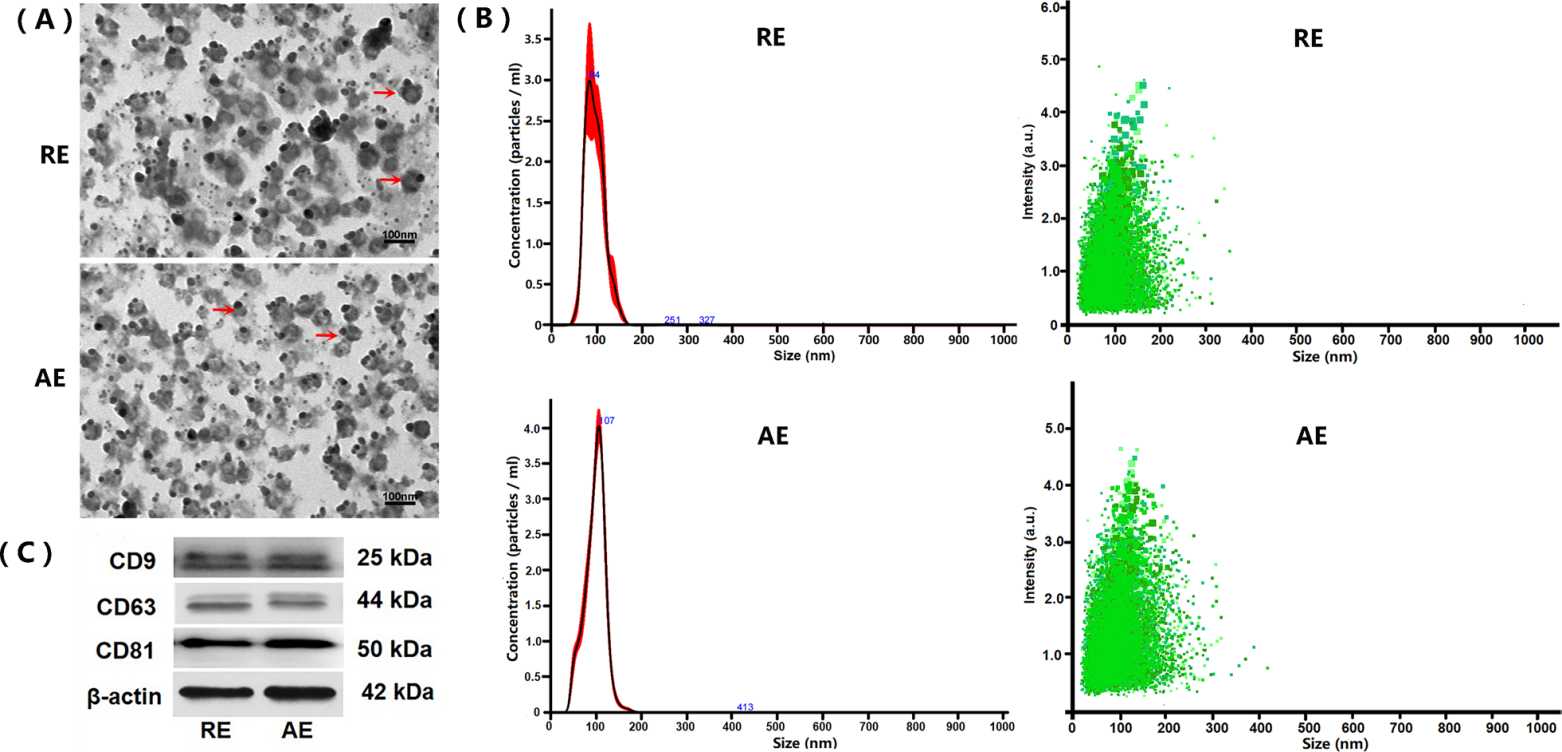
Characterization of resting and LPS-activated P815 mast cell-derived exosomes (designated as *RE* and *AE*, respectively). Exosomes were isolated from culture supernatants of resting and LPS-activated P815 mast cells, and then characterized by transmission electron microscopy (TEM), nanoparticle tracking analysis (NTA), and western blotting. **A** Representative TEM images showing disk-shaped structures of *RE* and *AE*. Red arrows indicate typical exosomal vesicles. Scale bar = 100 nm. **B** NTA analysis indicating the size distribution (nm) and particle concentration (particles/ml) on *RE* (2.51 ± 0.14 × 10^11^ particles/ml) and *AE* (6.17 ± 0.22 × 10^11^ particles/ml), respectively. **C** Western blotting analysis confirming positive expression of exosomal markers (CD9, CD63 and CD81) in both *RE* and *AE*

The morphology and size distribution of *RE* and *AE* were characterized by TEM, NTA, and western blotting. TEM imaging demonstrated that both *RE* and *AE* exhibited disk-shaped structures with diameters ranging from 20 to 100 nm (Fig. 1A). NTA revealed particle sizes of 21–124 nm for *RE* (peak: 84 nm) and 22–123 nm for *AE* (peak: 107 nm). A significant difference in exosome concentration was observed between *RE* and *AE* (*RE*: 2.51 ± 0.14 × 10^11^ particles/ml; *AE*: 6.17 ± 0.22 × 10^11^ particles/ml; *P* < 0.01) (Fig. 1B, C). Western blotting confirmed the presence of typical exosomal markers (CD9, CD63, and CD81) in both *RE* and *AE* (Fig. 1D). These results validate the successful isolation of exosomes from resting and LPS-activated P815 mast cells.

### *RE* and *AE* treatments exacerbated morbidity and mortality in *Pb*A-infected mice

**Fig. 2:**
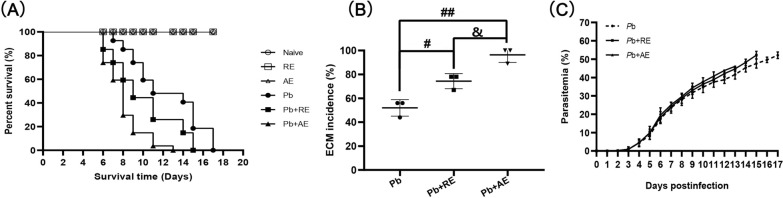
Effects of *RE* and *AE* on the changes in survival time, ECM incidence, and parasitemia in the *Plasmodium berghei* ANKA (*Pb*A)-infected mice. **A**, **B** Survival time and ECM incidence in the C57BL/6 mice (Naive, *RE*, *AE*: *n* = 4/group; *Pb*, *Pb*+*RE*, *Pb*+*AE*: *n* = 9/group) were monitored daily over 17 days across three independent experiments. **C** Parasitemia progression from day 3 post-infection was quantified via Giemsa-stained thin blood smears (tail vein), presented as iRBC percentage (iRBCs/total RBCs × 100%). Survival curves were analyzed by log-rank test; parasitemia dynamics were assessed by a time series analysis test. The experiment was independently repeated three times. ^#^*P* < 0.05 and ^##^*P* < 0.01 vs. the *Pb*A-infected ECM mice (*Pb* group). ^&^*P* < 0.05 and ^&&^*P* < 0.01 vs. the *RE*-treated ECM mice (*Pb*+*RE* group)

Naive, *RE*-treated, and *AE*-treated uninfected control mice exhibited 100% survival over the 17-day observation period (Fig. 2A). In contrast, *Pb*A-infected control mice (*Pb* group) died between 7–17 days p.i., with ~50% developing ECM. Mice receiving *Pb*A infection combined with *RE* (*Pb*+*RE* group) or *AE* (*Pb*+*AE* group) displayed accelerated mortality (6–15 days and 6–13 days p.i., respectively) and significantly elevated ECM incidence (*Pb*+*RE*: 75%; *Pb*+*AE*: 100%). Survival curve comparisons revealed significant differences between *Pb* vs. *Pb*+*RE* (*P* < 0.01) and *Pb*+*RE* vs. *Pb*+*AE* (*P* < 0.01). No significant intergroup differences in parasitemia were observed in *Pb*A-infected cohorts among *Pb*, *Pb*+*RE*, and *Pb*+*AE* groups (*P* > 0.05). Collectively, these results demonstrated that *RE* and *AE* treatments markedly exacerbated both morbidity and mortality outcomes during *Pb*A infection.

### *RE* and *AE* treatments exacerbate neuropathological damage in ECM mice

**Fig. 3:**
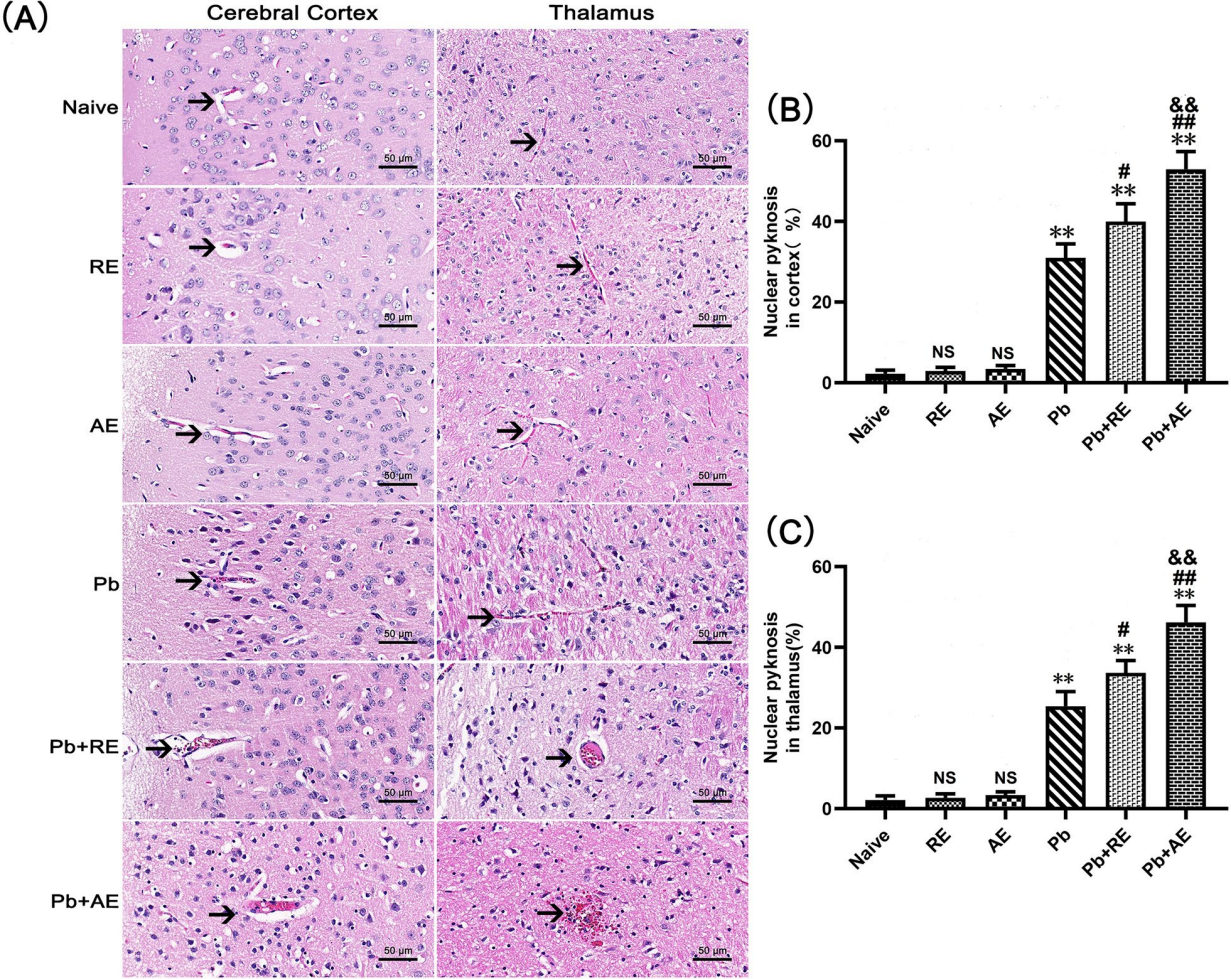
Effects of *RE* and *AE* on the brain histopathological changes in the ECM mice. **A** Representative images of histopathological changes by H&E staining in cerebral cortex (left) and thalamus (right) in uninfected mice from Naïve, *RE*, and *AE* groups, and ECM mice from the *Pb*, *Pb*+*RE*, and *Pb*+*AE* groups using a Leica DM2500B microscope at ×400 magnification. Arrows indicate typical blood vessels. **B**, **C** Quantification of pyknotic nuclei in cerebral cortex and thalamus was analyzed from mice in six groups by counting 20 non-overlapping fields/section (*n* = 6 mice/group) by two blinded investigators. Data are presented as mean percentage of pyknotic nuclei ± SD, and analyzed by unpaired Student’s *t*-tests between two groups. *P < 0.05 and **P < 0.01 vs. the uninfected control mice (Naïve group); ^#^*P* < 0.05 and ^##^*P* < 0.01 vs. the *Pb*A-infected ECM mice (*Pb* group). ^&^*P* < 0.05 and ^&&^*P* < 0.01 vs. the *RE*-treated ECM mice (*Pb*+*RE* group). NS: non-significant, *P* > 0.05, relative to Naïve mice

H&E staining was performed to assess brain pathology in ECM mice following *RE* or *AE* administration. Uninfected controls (Naive, *RE*, and *AE* groups) displayed intact neuronal architecture with regular cell arrangement, normal nuclear staining, and absence of inflammatory foci or iRBCs (Fig. 3A). In contrast, *Pb*A-infected ECM mice (*Pb* group) exhibited severe cerebral pathology, including cortical and thalamic iRBC sequestration, intravascular leukocyte infiltration, capillary occlusion, neuronal pyknosis, and disrupted cytoarchitecture. *RE* and *AE* treatments markedly exacerbated these pathological features in ECM mice, with *Pb*+*RE* and *Pb*+*AE* groups demonstrating increased iRBC accumulation (Fig. 3A), amplified inflammatory infiltrates, aggravated neuronal disorganization, and pronounced cerebral edema. Quantitative analysis revealed significantly higher proportions of pyknotic neurons in the cerebral cortex of *Pb*+*RE* mice compared to *Pb* controls (~30% vs. ~40%, *P* = 0.0441), with further elevation in *Pb*+*AE* mice (~40% vs. ~53%, *P* = 0.0069) (Fig. 3B). Similar trends were observed in thalamic regions. These data conclusively demonstrated that *RE* and *AE* exacerbated neuropathological progression during ECM.

### *RE* and *AE* treatments exacerbated BBB disruption in ECM mice

**Fig. 4:**
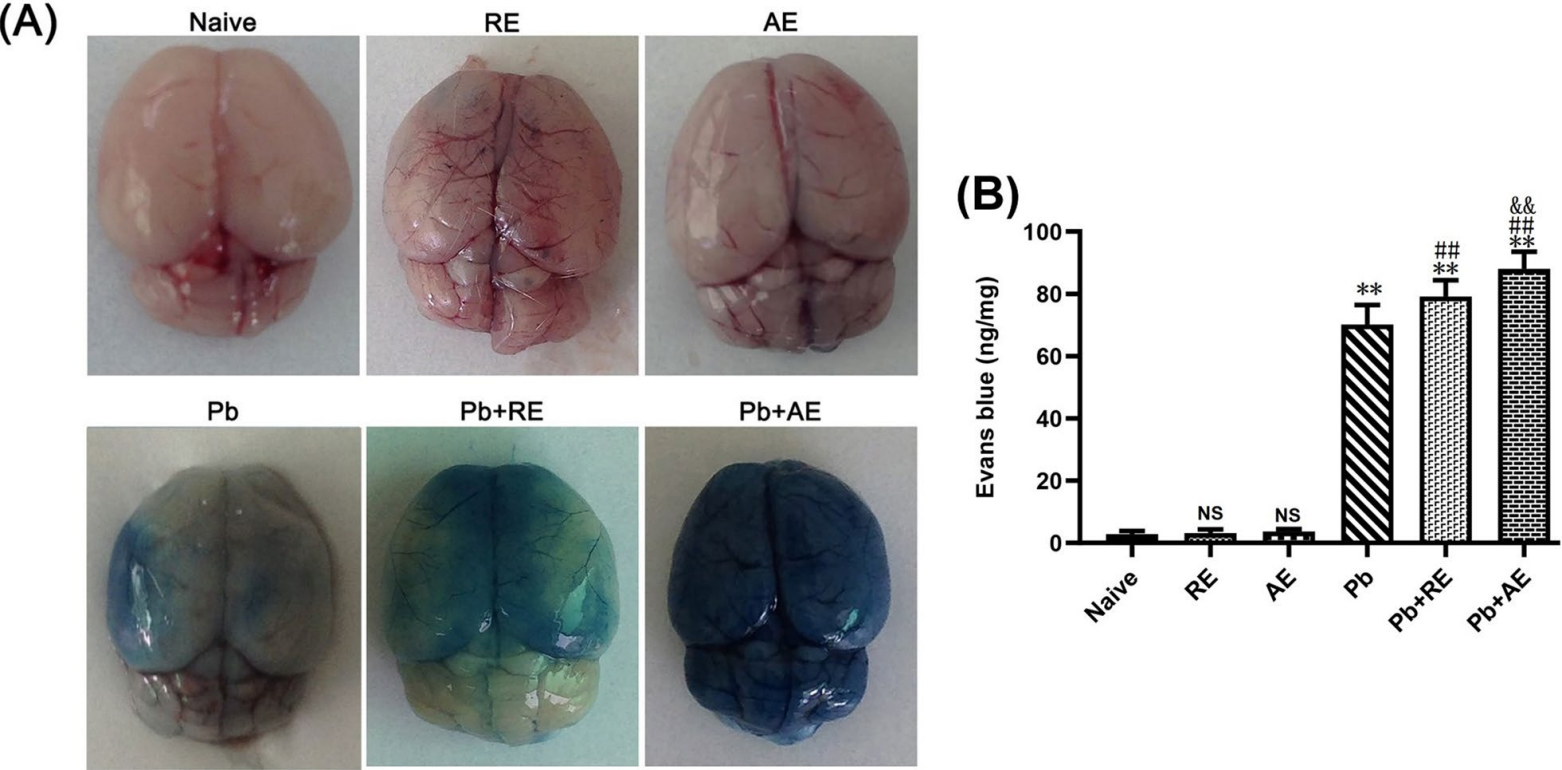
Effects of *RE* and *AE* on the changes in BBB integrity in the ECM mice. Evans blue (EB) dye was i.v. injected into uninfected mice from the Naïve, *RE*, and *AE* groups, and ECM from the *Pb*, *Pb*+*RE*, and *Pb*+*AE* groups. **A** Representative whole-brain photographs of EB extravasation in mice from six groups. **B** Quantification of EB extravasation in brain tissue of mice (*n* = 6/group, pooled from three independent experiments) was calculated from a standard curve (620 nm) and normalized to brain weight—ng (EB dye)/mg (brain weight). Data are presented as mean ± SD, and were analyzed by unpaired Student’s *t*-tests between two groups

EB extravasation assays revealed significant BBB integrity alterations in ECM mice following *RE* or *AE* administration. Uninfected naive, *RE*-treated, or *AE*-treated mice showed intact BBB integrity with no EB leakage into brain parenchyma (Fig. 4). ECM mice from the *Pb* group exhibited BBB disruption, evidenced by EB penetration (~70 ng/mg). *RE* (*Pb*+*RE* group: ~80 ng/mg; *P* < 0.01) and *AE* (*Pb*+*AE* group: 88 ng/mg; *P* < 0.01) treatments significantly increased EB levels compared to *Pb* controls. *AE*-treated ECM mice (*Pb*+*AE*) displayed ~10% higher EB accumulation than *RE*-treated mice (~88 vs. ~80 ng/mg; *P* < 0.01). These findings demonstrated that *RE* and *AE* treatments exacerbated BBB disruption during ECM progression.

### *RE* and *AE* treatments exerted extensive neuroinflammation in ECM mice

**Fig. 5:**
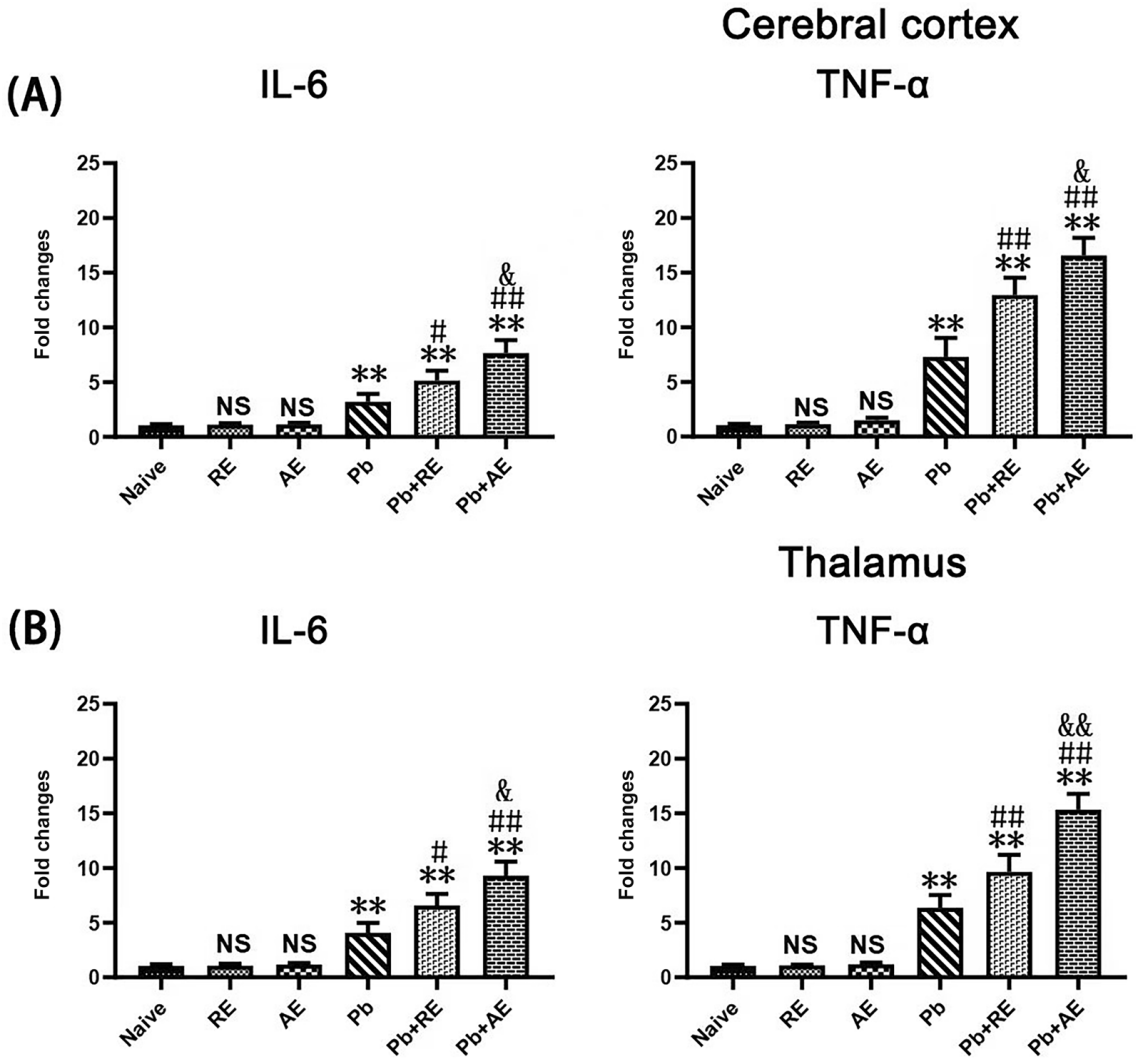
Effects of RE and AE on the changes in neuroinflammation response in the ECM mice. (**A**,** B**) Total RNA was extracted from cerebral cortex (Upper) and thalamus (Lower) at uninfected mice from Naïve (*n* = 6),* RE* (*n* = 6), and* AE* (*n* = 6) groups, and ECM from* Pb* (*n* = 6), Pb+RE (*n* = 6), and Pb+AE (*n* = 6) groups. The mRNA levels of IL-6, TNF-α,and IL-1β were determined using qPCR and 2−ΔΔCT methods with β-actin as the endogenous reference gene. Independent-samples t-tests were conducted to assess differences between two groups. Data were presented as mean ± SD.^*^ P < 0.05 and ^**^ P < 0.01 vs. the uninfected control mice (Naïve group); ^#^ P < 0.05 and ^##^ P < 0.01 vs. the PbA-infectedECM mice (Pb group). ^&^* P* < 0.05 and ^&&^* P* < 0.01 vs. the RE-treated ECM mice (Pb+RE group). NS = non-significant,*P* > 0.05, relative to Naïve mice.

qPCR analysis assessed IL-6, TNF-α, and IL-1β mRNA levels to evaluate *RE* and *AE* effects on neuroinflammation in ECM mice. Cortical IL-6, TNF-α, and IL-1β levels remained unchanged in uninfected *RE*- or *AE*-treated mice compared to naive controls (*P* > 0.05; Fig. 5). ECM mice from the *Pb* group exhibited elevated cortical IL-6 (*P* < 0.01), TNF-α (*P* < 0.01), and IL-1β (*P* < 0.01) mRNA levels versus Naive mice. *RE* (*Pb*+*RE* group) and *AE* (*Pb*+*AE* group) treatments further increased cortical IL-6 (*RE*: *P* < 0.01; *AE*: *P* < 0.01), TNF-α (*RE*: *P* < 0.01; *AE*: *P* < 0.01), and IL-1β (*RE*: *P* < 0.05; *AE*: *P* < 0.01) levels compared to *Pb* controls. AE-treated ECM mice (*Pb*+*AE*) showed higher cortical IL-6 (*P* < 0.05), TNF-α (*P* < 0.01), and IL-1β (*P* < 0.05) levels than *RE*-treated ECM mice (*Pb*+*RE*). Thalamic cytokine patterns mirrored cortical trends. These results demonstrated that *RE* and *AE* treatments amplified pro-inflammatory responses in ECM mice.

### *RE* and* AE* treatments exacerbate neuronal injury and apoptosis in ECM mice

**Fig. 6:**
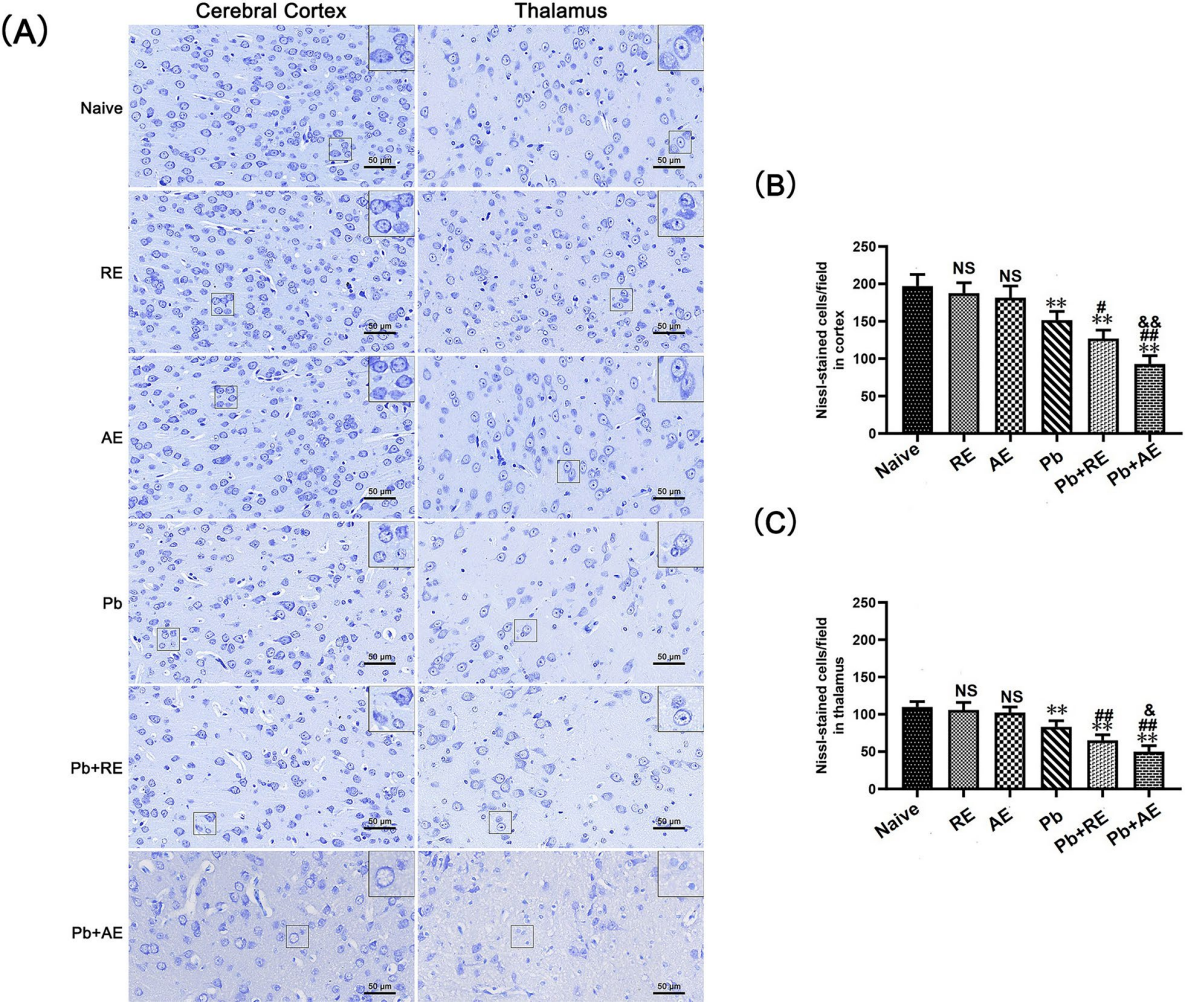
Effects of *RE* and *AE* on the changes in number of Nissl-stained cells in the ECM mice. **A** Representative Nissl-stained micrographs of cerebral cortex (left) and thalamus (right) in uninfected mice from the Naïve, *RE*, and *AE* groups, and ECM mice from the *Pb*, *Pb*+*RE*, and *Pb*+*AE* groups using a Leica DM2500B microscope at ×400 magnification. Square insets show Nissl-stained cells at a higher magnification (×1000). **B**, **C** Quantification of intact Nissl^+^ cells in cerebral cortex (**B**) and thalamus (**C**) were analyzed from mice in six groups by counting 20 non-overlapping fields/section (*n* = 6 mice/group). Independent-samples *t*-tests were conducted to assess differences between two groups. Data are presented as mean ± SD. *P < 0.05 and **P < 0.01 vs. the uninfected control mice (Naïve group); ^#^*P* < 0.05 and ^##^*P* < 0.01 vs. the *Pb*A-infected ECM mice (*Pb* group). ^&^*P* < 0.05 and ^&&^*P* < 0.01 vs. the *RE*-treated ECM mice (*Pb*+*RE* group). NS: non-significant, *P* > 0.05, relative to Naïve mice

**Fig.7:**
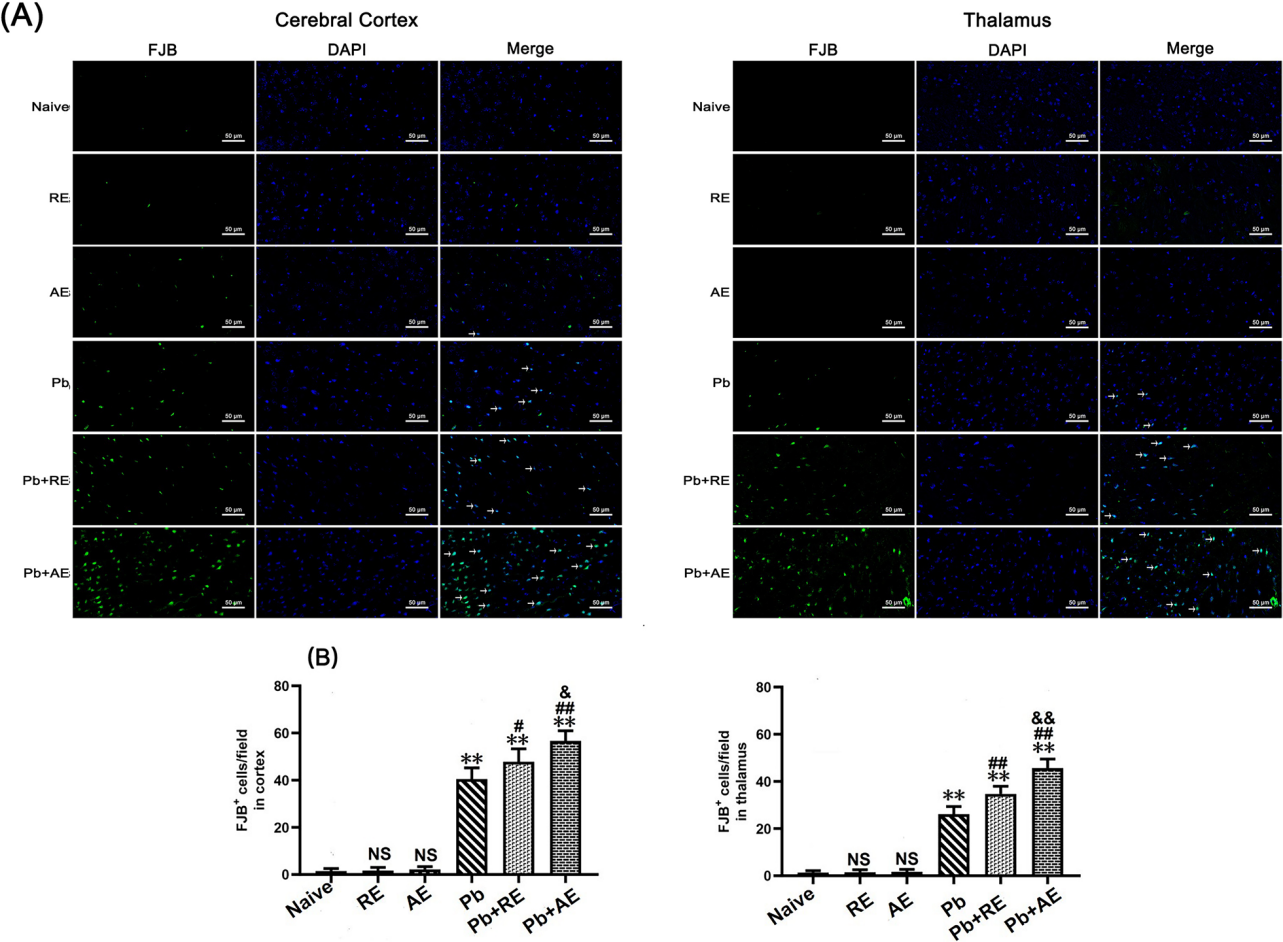
Effects of RE and AE on the changes in number of FJB-stained neurons in the ECM mice. (**A**) Representative fluorescent micrographs of FJB-stained neurons of cerebral cortex (Left) and thalamus (Right) at uninfected mice from Naïve, RE, and AE groups, and ECM mice from Pb, Pb+RE, and Pb+AE groups. Positive dual FJB+-DAPI+ stained cells were illustrated by green fluorescence (arrows) and captured by a Zeiss LSM780 confocal microscope at 400 × magnification. (**B**) Quantification of intact FJB+ cells in cerebral cortex (Left) and thalamus (Right) were analyzed from mice at six groups by accounting 20 non-overlapping fields/section (*n* = 6 mice/group). Independent-samples t-tests were conducted to assess differences between two groups. Data were presented as mean± SD. ^*^P < 0.05 and ^**^P < 0.01 vs the uninfected control mice (Naïve group); ^#^*P* < 0.05 and ^##^*P* < 0.01 vs. the PbA-infected ECM mice (Pb group). ^&^*P* <0.05 and ^&&^*P* < 0.01 vs. the RE-treated ECM mice (Pb+RE group). NS = non-significant,*P* > 0.05, relative to Naïvemice

Given that reduced Nissl body numbers indicate neuronal dysfunction, we evaluated neuronal injury in ECM mice using Nissl staining. In Naive, *RE*-treated, or *AE*-treated uninfected mice, Nissl bodies were evenly distributed in cortical neurons (Fig. 6). ECM mice from the *Pb* group exhibited a significant reduction in cortical Nissl-positive neurons compared to Naive controls (*P* < 0.01). Both *RE* (~127 vs. ~151 cells/field; *P* < 0.05) and *AE* (~93 vs. ~151 cells/field; *P* < 0.01) treatments further decreased cortical Nissl-positive neuron counts relative to the *Pb* group. Notably, *AE*-treated ECM mice (*Pb*+*AE* group) showed fewer cortical Nissl-positive neurons than *RE*-treated ECM mice (~93 vs. ~127 cells/field; *P* < 0.01), with similar thalamic trends observed. FJB staining revealed minimal cortical neuronal injury in Naive, *RE*-treated, or *AE*-treated uninfected mice (Fig. 7). Cortical FJB-positive neurons increased in the *Pb*+*RE* group compared to *Pb* controls (~48 vs. ~40 cells/field; *P* < 0.05) and were further elevated in the *Pb*+*AE* group (~57 vs. ~48 cells/field; *P* < 0.05). Thalamic FJB-positive neurons followed comparable patterns. Collectively, these findings demonstrated that *RE* and *AE* treatments exacerbated neuronal injury in ECM mice.

**Fig. 8:**
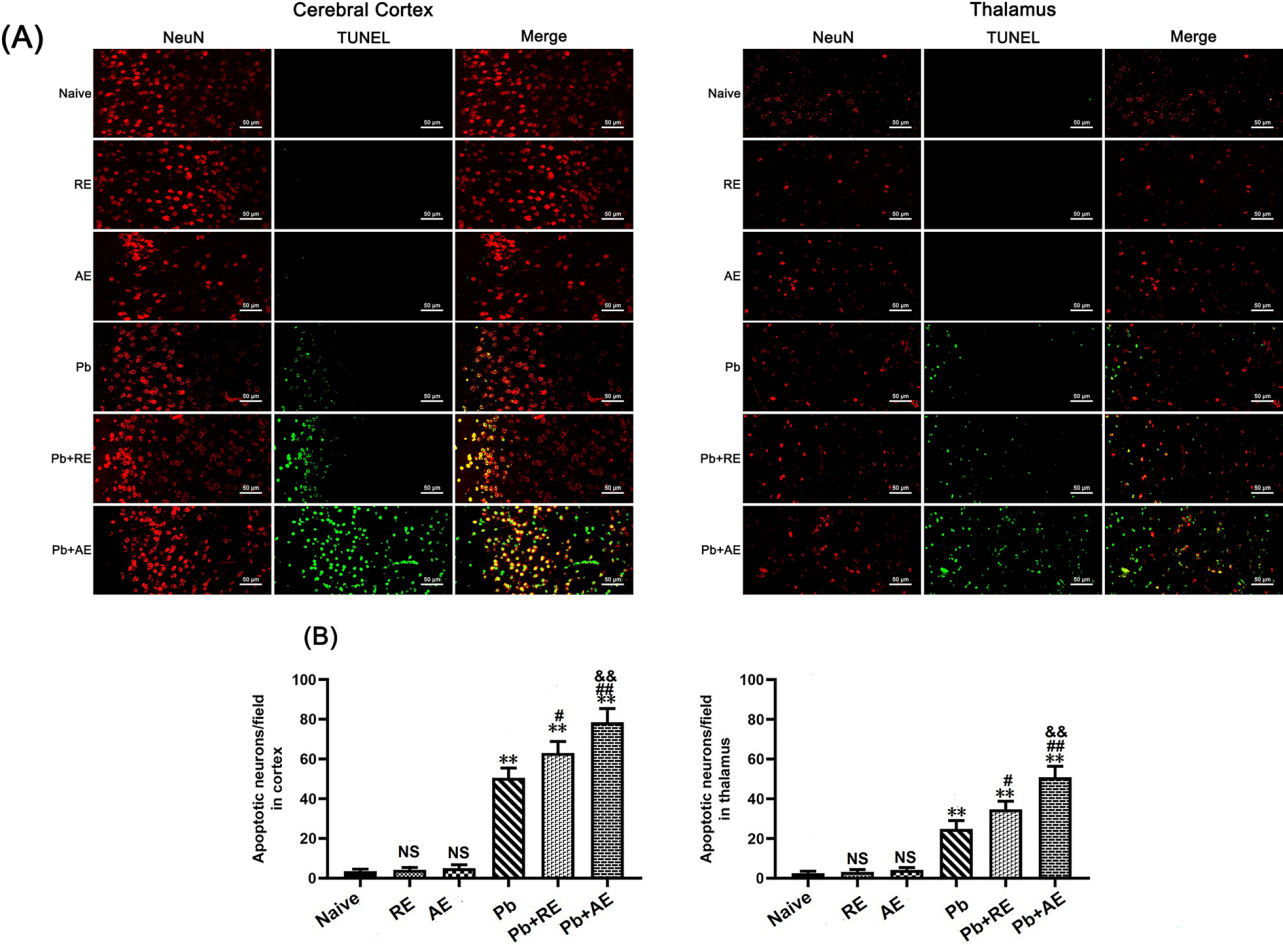
Effects of *RE* and *AE* on the changes in number of dual immunofluorescence staining TUNEL^+^-NeuN^+^ neurons in the ECM mice. **A** Representative dual immunofluorescence micrographs of TUNEL^+^-NeuN^+^ neurons in the cerebral cortex (left) and thalamus (right) in uninfected mice from Naïve, *RE*, and *AE* groups, and ECM mice from *Pb*, *Pb*+*RE*, and *Pb*+*AE* groups. Positive dual TUNEL^+^-NeuN^+^ stained cells were illustrated by yellow fluorescence and captured by a Zeiss LSM780 confocal microscope at ×400 magnification. **B** Quantification of TUNEL^+^-NeuN^+^ neurons in cerebral cortex and thalamus was analyzed from mice in six groups by counting 20 non-overlapping fields/section (*n* = 6 mice/group). Independent-samples *t*-tests were conducted to assess differences between two groups. Data are presented as mean ± SD. *P < 0.05 and **P < 0.01 vs. the uninfected control mice (Naïve group); ^#^*P* < 0.05 and ^##^*P* < 0.01 vs. the *Pb*A-infected ECM mice (*Pb* group). ^&^*P* < 0.05 and ^&&^*P* < 0.01 vs. the *RE*-treated ECM mice (*Pb*+*RE* group). NS: non-significant, *P* > 0.05, relative to Naïve mice

Next, TUNEL^+^-NeuN^+^ double immunofluorescence staining was performed to assess neuronal apoptosis in ECM mice. Minimal TUNEL^+^-NeuN^+^ neurons were observed in Naive, *RE*-treated, or *AE*-treated uninfected mice (Fig. 8). ECM mice from the *Pb* group showed significantly elevated cortical TUNEL^+^-NeuN^+^ neurons compared to Naive controls. *RE* (~63 vs. ~50 cells/field; *P* < 0.05) and *AE* (~79 vs. ~50 cells/field; *P* < 0.01) treatments further increased cortical TUNEL^+^-NeuN^+^ neuron counts relative to *Pb* controls. Notably, *AE*-treated ECM mice *(Pb*+*AE* group) exhibited higher cortical TUNEL^+^-NeuN^+^ neurons than *RE*-treated mice (~79 vs. ~63 cells/field;* P* < 0.05). Similar thalamic TUNEL^+^-NeuN^+^ neuron patterns emerged post-treatment. These results demonstrated that *RE* and *AE* treatments augmented neuronal apoptosis during ECM progression.

### *RE *and *AE* treatments amplified ER stress in ECM mice neurons

**Fig. 9:**
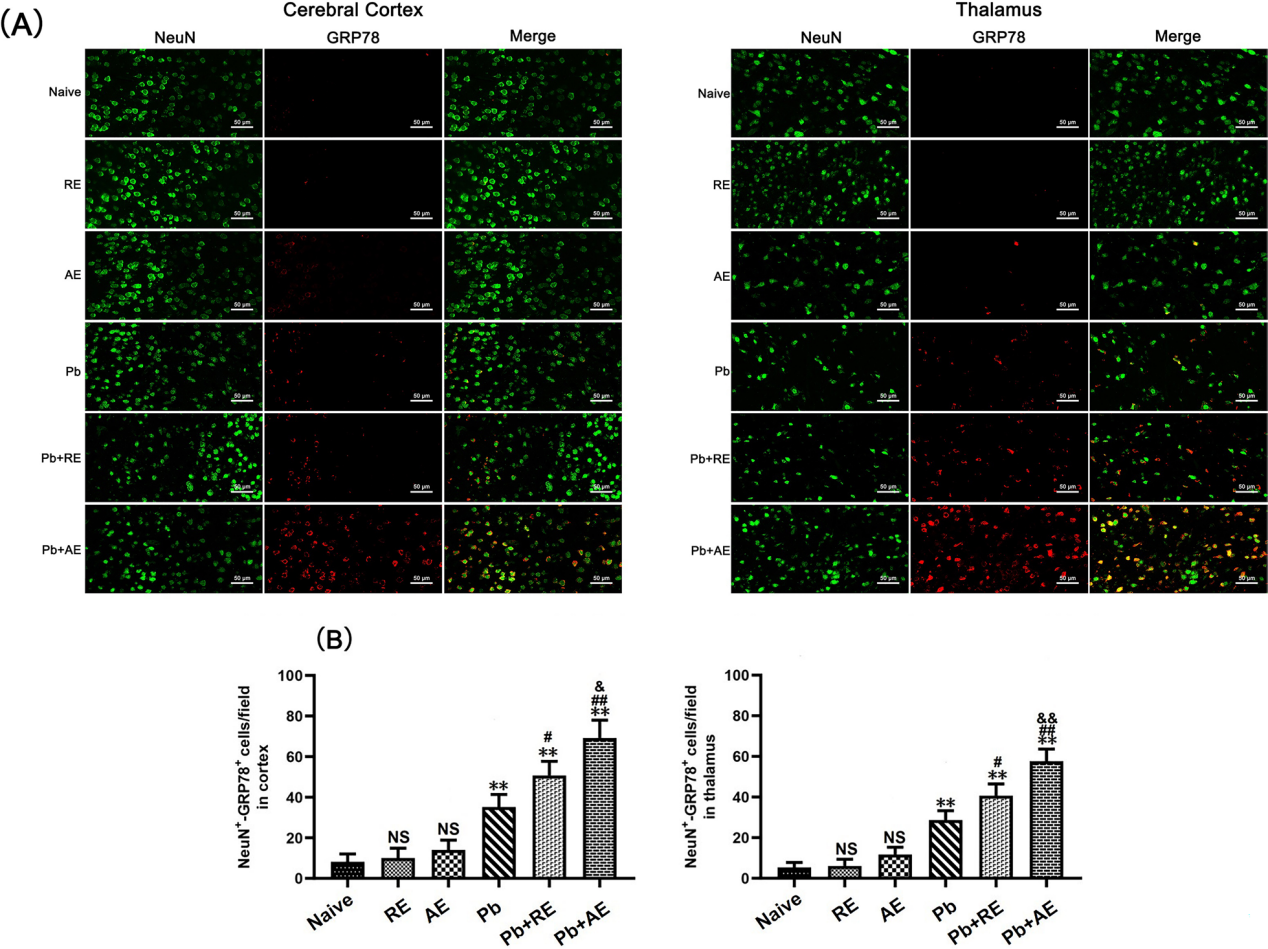
Effects of *RE* and *AE* on the changes in number of dual immunofluorescence NeuN^+^-GRP78^+^ neurons in the ECM mice. **A** Representative dual immunofluorescence micrographs of NeuN^+^-GRP78^+^ neurons in the cerebral cortex (left) and thalamus (right) in uninfected mice from Naïve, *RE*, and *AE* groups, and ECM mice from *Pb*, *Pb*+*RE*, and *Pb*+*AE* groups. Positive dual TUNEL^+^-NeuN^+^ stained cells were illustrated by yellow fluorescence and captured by a Zeiss LSM780 confocal microscope at ×400 magnification. **B** Quantification of NeuN^+^-GRP78^+^ neurons in cerebral cortex and thalamus was analyzed from mice in six groups by counting 20 non-overlapping fields/section (*n* = 6 mice/group). Independent-samples *t*-tests were conducted to assess differences between two groups. Data are presented as mean ± SD. *P < 0.05 and **P < 0.01 vs. the uninfected control mice (Naïve group); ^#^*P* < 0.05 and ^##^*P* < 0.01 vs. the *Pb*A-infected ECM mice (*Pb* group). ^&^*P* < 0.05 and ^&&^*P* < 0.01 vs. the *RE*-treated ECM mice (*Pb*+*RE* group). NS: non-significant, *P* > 0.05, relative to Naïve mice

**Fig. 10:**
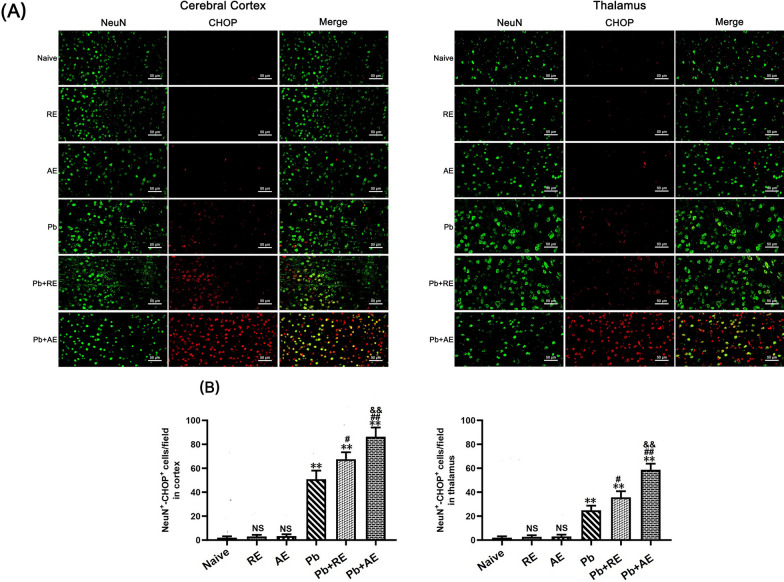
Effects of *RE* and *AE* on the changes in number of dual immunofluorescence NeuN^+^-CHOP^+^ neurons in the ECM mice. **A** Representative dual immunofluorescence micrographs of NeuN^+^-CHOP^+^ neurons in the cerebral cortex (left) and thalamus (right) in uninfected mice from Naïve, *RE*, and *AE* groups, and ECM mice from *Pb*, *Pb*+*RE*, and *Pb*+*AE* groups. Positive dual TUNEL^+^-NeuN^+^ stained cells were illustrated by yellow fluorescence and captured by a Zeiss LSM780 confocal microscope at ×400 magnification. **B** Quantification of NeuN^+^-CHOP^+^ neurons in cerebral cortex and thalamus was analyzed from mice in six groups by counting 20 non-overlapping fields/section (*n* = 6 mice/group). Independent-samples *t*-tests were conducted to assess differences between two groups. Data are presented as mean ± SD. *P < 0.05 and **P < 0.01 vs. the uninfected control mice (Naïve group); ^#^*P* < 0.05 and ^##^*P* < 0.01 vs. the *Pb*A-infected ECM mice (*Pb* group). ^&^*P* < 0.05 and ^&&^*P* < 0.01 vs. the *RE*-treated ECM mice (*Pb*+*RE* group). NS: non-significant, *P* > 0.05, relative to Naïve mice

**Fig. 11:**
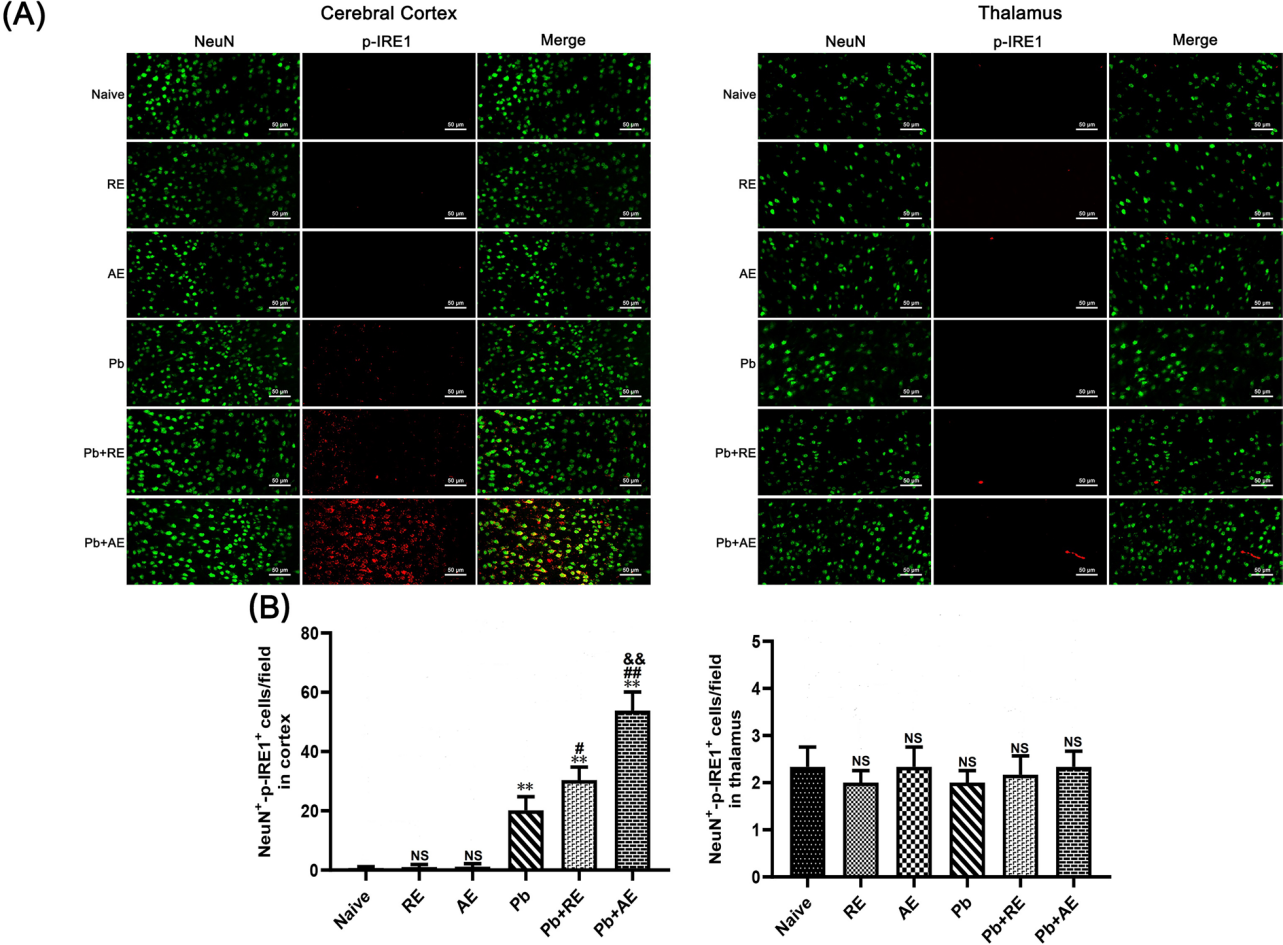
Effects of *RE* and *AE* on the changes in number of dual immunofluorescence NeuN^+^-p-IRE1^+^ neurons in the ECM mice. **A** Representative dual immunofluorescence micrographs of NeuN^+^-p-IRE1^+^ neurons in the cerebral cortex (left) and thalamus (right) in uninfected mice from Naïve, *RE*, and *AE* groups, and ECM mice from *Pb*, *Pb*+*RE*, and *Pb*+*AE* groups. Positive dual TUNEL^+^-NeuN^+^ stained cells were illustrated by yellow fluorescence and captured by a Zeiss LSM780 confocal microscope at ×400 magnification. **B** Quantification of NeuN^+^-p-IRE1^+^ neurons in cerebral cortex and thalamus was analyzed from mice in six groups by counting 20 non-overlapping fields/section (*n* = 6 mice/group). Independent-samples *t*-tests were conducted to assess differences between two groups. Data are presented as mean ± SD. *P < 0.05 and **P < 0.01 vs. the uninfected control mice (Naïve group); ^#^*P* < 0.05 and ^##^*P* < 0.01 vs. the *Pb*A-infected ECM mice (*Pb* group). ^&^*P* < 0.05 and ^&&^*P* < 0.01 vs. the *RE*-treated ECM mice (*Pb*+*RE* group). NS: non-significant, *P* > 0.05, relative to Naïve mice

**Fig. 12:**
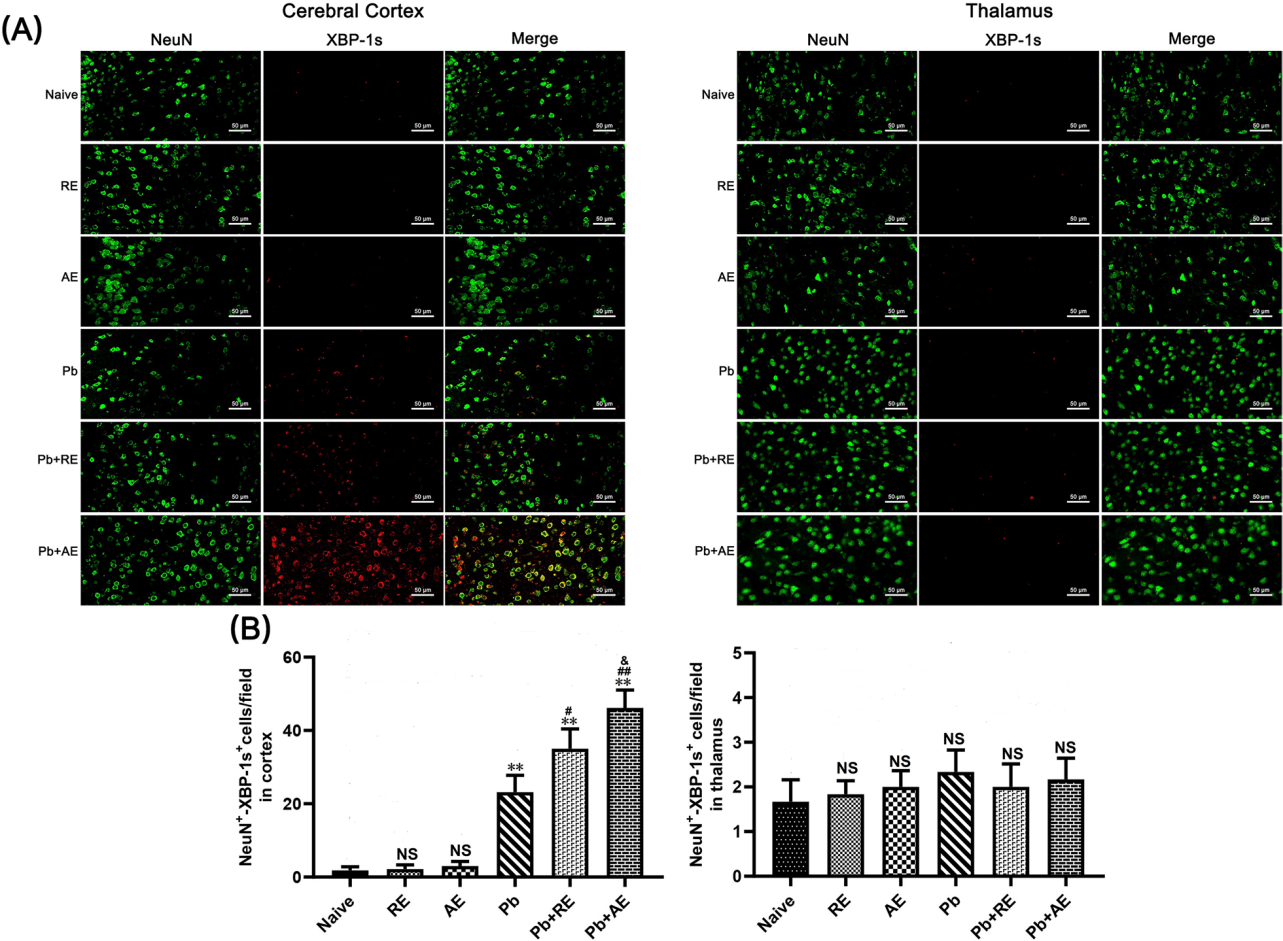
Effects of *RE* and *AE* on the changes in number of dual immunofluorescence NeuN^+^-XBP-1s^+^ neurons in the cerebral cortex of the ECM mice. **A** Representative dual immunofluorescence micrographs of NeuN^+^-XBP-1s^+^ neurons in the cerebral cortex (left) and thalamus (right) in uninfected mice from Naïve, *RE*, and *AE* groups, and ECM mice from *Pb*, *Pb*+*RE*, and *Pb*+*AE* groups. Positive dual TUNEL^+^-NeuN^+^ stained cells were illustrated by yellow fluorescence and captured by a Zeiss LSM780 confocal microscope at ×400 magnification. **B** Quantification of NeuN^+^-XBP-1s^+^ neurons in cerebral cortex and thalamus was analyzed from mice in six groups by counting 20 non-overlapping fields/section (*n* = 6 mice/group). Independent-samples *t*-tests were conducted to assess differences between two groups. Data are presented as mean ± SD. *P < 0.05 and **P < 0.01 vs. the uninfected control mice (Naïve group); ^#^*P* < 0.05 and ^##^*P* < 0.01 vs. the *Pb*A-infected ECM mice (*Pb* group). ^&^*P* < 0.05 and ^&&^*P* < 0.01 vs. the *RE*-treated ECM mice (*Pb*+*RE* group). NS: non-significant, *P* > 0.05, relative to Naïve mice

We analyzed ER stress-related proteins (GRP78, CHOP, p-IRE1, and XBP-1s) in cortical and thalamic neurons of ECM mice using dual immunofluorescence staining. Minimal NeuN^+^-GRP78^+^, NeuN^+^-CHOP^+^, NeuN^+^-p-IRE1^+^, and NeuN^+^-XBP-1s^+^ neurons were observed in Naive, *RE*-treated, or *AE*-treated uninfected mice (Figs. 9, 10, 11 and 12). In contrast, ECM mice from the *Pb* group exhibited significant increases in cortical NeuN^+^-GRP78^+^ (*P* < 0.01), NeuN^+^-CHOP^+^ (*P* < 0.01), NeuN^+^-p-IRE1^+^ (*P* < 0.01), and NeuN^+^-XBP-1s^+^ (*P* < 0.01) neurons, along with thalamic NeuN^+^-GRP78^+^ (*P* < 0.01) and NeuN^+^-CHOP^+^ (*P* < 0.01) neurons, compared to Naive controls. *RE* treatment (*Pb*+*RE* group) further elevated cortical NeuN^+^-GRP78^+^ (~51 vs. ~35 cells/field; *P* < 0.05), NeuN^+^-CHOP^+^ (~67 vs. ~51 cells/field; *P* < 0.05), NeuN^+^-p-IRE1^+^ (~30 vs. ~20 cells/field; *P* < 0.05), and NeuN^+^-XBP-1s^+^ (~35 vs. ~23 cells/field; *P* < 0.05) neurons relative to the *Pb* group. Strikingly, *AE* treatment (*Pb*+*AE* group) induced higher cortical NeuN^+^-GRP78^+^ (~69 vs. ~51 cells/field; *P* < 0.01), NeuN^+^-CHOP^+^ (~86 vs. ~51 cells/field; *P* < 0.01), NeuN^+^-p-IRE1^+^ (~54 vs. ~30 cells/field; *P* < 0.01), and NeuN^+^-XBP-1s^+^ (~46 vs. ~35 cells/field; *P* < 0.05) neuron counts compared to *Pb*+*RE*. However, *RE* and *AE* treatments did not alter thalamic NeuN^+^-p-IRE1^+^ or NeuN^+^-XBP-1s^+^ neuron numbers in ECM mice. These findings demonstrated that *RE* and *AE* potentiated neuronal ER stress in ECM pathogenesis, with *AE* exhibiting greater ER stress-inducing capacity than *RE*.

### *RE* and *AE* treatments increased the mRNA level of ER stress-related factors (GRP78, CHOP, and XBP-1) in neuronal HT-22 cells in vitro

**Fig. 13:**
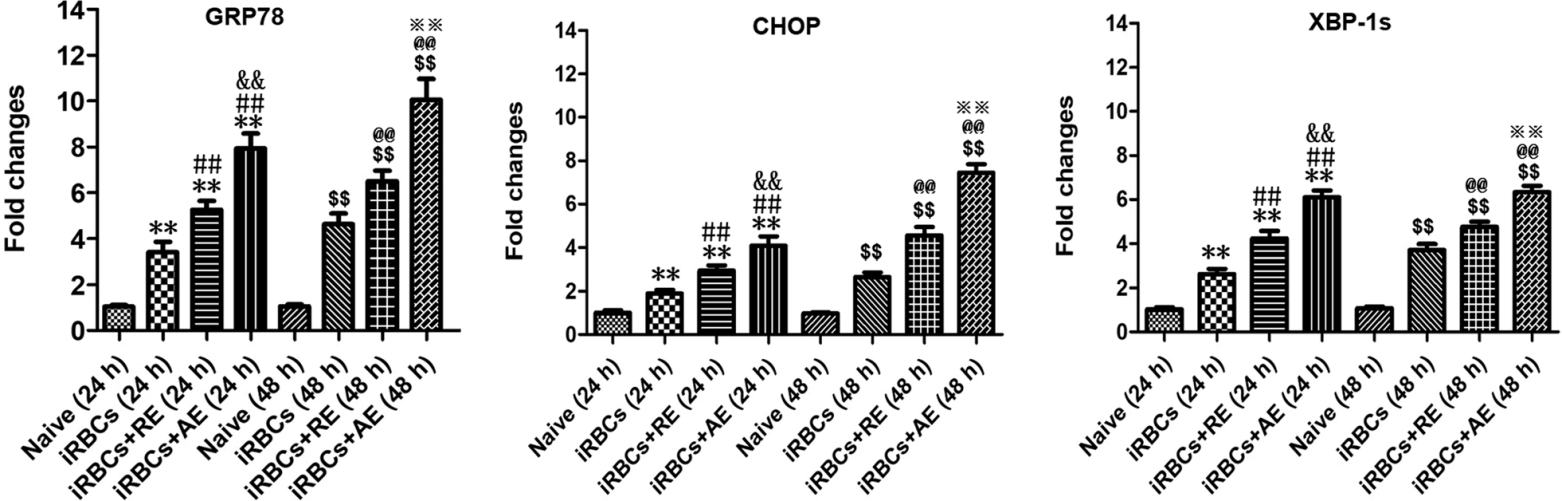
Effects of *RE* and *AE* on the changes in mRNA levels of ER stress-related factors (GRP78, CHOP, and XBP-1s) in the iRBC-stimulated neuronal HT-22 cells in vitro. The neuronal HT-22 cells were co-cultured with 5.0 × 10^6^ iRBCs for 6 h, followed by the addition of 100 μl ice-cold PBS buffer without or with *RE* (100 µg/ml) and *AE* (100 µg/ml) for 24 h and 48 h, respectively. The mRNA levels of target genes (GRP78, CHOP, and XBP-1s) were calculated using SYBR Green qPCR Master Mix and analyzed using the 2^−ΔΔCT^ method with β-actin as the endogenous reference gene. ^*^P < 0.05 and ^**^P < 0.01 vs. the Naive group for 24 h; ^#^*P* < 0.05 and ^##^*P* < 0.01 vs. the iRBC-stimulated HT-22 cell group at 24 h; ^&^*P* < 0.05 and ^&&^*P* < 0.01 vs. the iRBC-stimulated HT-22 cells targeted with *RE* treatment at 24 h; ^$^*P* < 0.05 and ^$$^*P* < 0.01 vs. the Naive group for 48 h; ^@^*P* < 0.05 and ^@@^*P* < 0.01 vs. the iRBC-stimulated HT-22 cell group at 48 h; ^※^*P* < 0.05 and ^※※^*P* < 0.01 vs. the iRBC-stimulated HT-22 cells targeted with *RE* treatment at 48 h; All data were taken from four independent experiments, presented as mean ± SD, and analyzed using independent-samples *t*-tests between two groups

As shown in Fig. 13, iRBC-stimulated neuronal HT-22 cells exhibited a significant increase in the mRNA levels of GRP78, CHOP, and XBP-1 compared to the blank group at 24 h and 48 h of co-culture (*P* < 0.01 for all comparisons). Both *RE* and *AE* treatments further elevated mRNA levels of GRP78 (*P* < 0.01 for *RE*, *P* < 0.01 for *AE*), CHOP (*P* < 0.01 for *RE*, *P* < 0.01 for *AE*), and XBP-1 (*P* < 0.05 for *RE*, *P* < 0.01 for *AE*) in the iRBC-stimulated HT-22 cells at both 24 h and 48 h of co-culture, respectively. Notably, *RE*-treated iRBC-stimulated HT-22 cells showed higher mRNA levels of GRP78 (~45% and ~47% increase, *P* < 0.01), CHOP (~33% and ~60% increase, *P* < 0.01), and XBP-1 (~50% and ~54% increase, *P* < 0.01) than *AE*-treated cells at 24 h and 48 h of co-culture, respectively. These findings demonstrate that *RE* and *AE* treatment induces ER stress responses in HT-22 cells in vitro.

### Distinct miRNA profiles underlie* RE*- and *AE*-mediated ER stress modulation

Given that microRNAs have emerged as vital regulators in ER stress responses [39], we performed miRNA sequencing (miRNA-seq) to compare microRNA expression profiles between *RE* and *AE*. The sequencing data revealed 128 differentially expressed microRNAs (56 upregulated and 72 downregulated in *AE* relative to *RE*; Supplementary Fig. 1). Notably, three microRNAs (miR-330-3p, miR-185-5p, and miR-379-5p), which were downregulated in *AE* compared to *RE*, have been reported to directly target ER stress-related proteins GRP78 and XBP-1s [40–42]. Conversely, six microRNAs (miR-155-5p, miR-423-3p, miR-187-3p, miR-29c-3p, miR-188-5p, and miR-192-5p) showed significant enrichment in *AE* versus *RE* and have been implicated in the indirect upregulation of ER stress-related proteins including GRP78 and CHOP [43–48].

## Discussion

Neuronal injury is a central driver of CM pathogenesis and its long-term neurological sequelae [4, 49, 50]. Post-mortem analyses of human CM patients revealed elevated ER stress markers (GRP78, CHOP) in brain neurons [51], with subsequent studies confirming ER stress as a critical mediator of neuronal death in ECM models [30]. Mast cell activation, known to amplify ER stress [31, 32], has been implicated in neurological disorders [52, 53], while clinical evidence highlights extracellular vesicles (e.g., *P. falciparum*-derived exosomes) as key contributors to endothelial dysfunction and neuroinflammation in CM [54, 55]. Emerging studies suggest mast cell–neuron cross-talk via exosomal transfer of bioactive molecules [20, 32, 56], exemplified by mast cell-derived exosomes interacting with sensory neurons through substance P-mediated activation of Mas-related G protein-coupled receptors [57] or neuronal uptake of mast cell extracellular vesicles [58]. Elevated mast cell degranulation in *P. falciparum*-infected patients correlates with parasitemia and disease severity [14], implicating mast cell-derived exosomes in CM neuropathology. Building on evidence that exosomes mediate mast cell–neuron communication and exacerbate ECM severity [15, 25], we hypothesized that activated mast cell-derived exosomes induce neuronal injury via ER stress. Exosomes from resting and LPS-activated P815 mast cells (*RE*/*AE*) were isolated and characterized using TEM, NTA, and western blotting, following methodologies from prior neuroinflammation studies [36]. Intravenous administration of a lower exosome dose (50 µg/mouse, vs. 200 µg in earlier BBB disruption work [25]) to ECM mice increased morbidity/mortality and exacerbated BBB disruption, neuronal apoptosis, pro-inflammatory responses, and neuronal ER stress marker expression (GRP78, CHOP, XBP-1). Divergent EB extravasation patterns compared to earlier reports [59–61] likely stem from methodological variations (e.g., dosage, parasite strain, host genetics). In vitro, *RE*/*AE* upregulated ER stress markers in iRBC-stimulated HT-22 neurons, mechanistically linking mast cell-derived exosomes to ER stress-driven neuronal injury in CM. These findings underscore mast cell–neuron interactions as pivotal therapeutic targets for mitigating CM-related neuropathology.

Neuroinflammation following BBB disruption drove neuronal injury in CM, contributing to reversible or irreversible neurological sequelae in pediatric CM patients and ECM models [6, 62]. Pathological progression of CM/ECM involved an imbalance between pro- and anti-inflammatory responses [63], with excessive pro-inflammatory cytokines (e.g., interferon gamma [IFN-γ], TNF-α, IL-6, IL-1α) exacerbating immunopathology [64]. In this study, ECM mice exhibited elevated IL-6, TNF-α, and IL-1β mRNA levels in brain tissue, accompanied by reduced Nissl-stained neurons and increased FJB-positive neurons, reflecting neuroinflammation-associated neuronal injury. These findings align with prior reports linking upregulated pro-inflammatory cytokines (IL-6, TNF-α, IL-1β, IFN-γ) in ECM brains to dysregulated immune responses and neuronal damage [65]. Mast cells, critical contributors to BBB disruption, neuroinflammation, and neuronal injury [66, 67], influenced CM severity [15, 25, 68], although their exosome-mediated interactions with neuroinflammatory pathways remain understudied. Intravenous administration of exosomes from resting (*RE*) or activated (*AE*) P815 mast cells significantly increased cortical and thalamic IL-6, TNF-α, and IL-1β mRNA levels in ECM mice, correlating with neuronal loss (reduced Nissl staining) and neurodegeneration (elevated FJB staining). This parallels reports of mast cell activation exacerbating microglia-mediated neuronal injury via TNF-α/IL-6 upregulation [69], while mast cell tryptase inhibition attenuates neuroinflammation and improves outcomes in neurological injury models [70]. Our data collectively demonstrated that mast cell-derived exosomes could amplify neuroinflammatory cascades and neuronal injury during ECM progression, reinforcing their pathogenic role in CM-related neuropathology.

Excessive ER stress, triggered by sensor activation (IRE1/sXBP1, PERK/EIF2, and ATF6), played a critical role in neuronal injury and pathology [28]. Similarly, ECM mice exhibited ER stress-induced neuronal injury/death in brain tissue, accompanied by upregulated PERK, IRE1, and ATF6 expression [30]. Given the emerging recognition of mast cell–ER stress interactions in neurological diseases [32], we hypothesized that mast cell-derived exosomes could exacerbate ECM neuronal injury by modulating ER stress. In this study, treatment with mast cell-derived exosomes—particularly *AE*—significantly elevated expression of IRE1 pathway-related ER stress markers (GRP78, CHOP, p-IRE1, and XBP-1s) in cortical neurons of *Pb*A-infected ECM mice. This was evidenced by increased numbers of NeuN^+^-GRP78^+^, NeuN^+^-CHOP^+^, NeuN^+^-p-IRE1^+^, and NeuN^+^-XBP-1s^+^ dual-positive neurons following *RE* and *AE* administration. Notably, *RE* and *AE* also upregulated mRNA levels of GRP78, CHOP, and XBP-1s in iRBC-stimulated HT-22 neuronal cells in vitro. However, our data revealed that treatments with *RE* and *AE* significantly upregulated ER stress markers (p-IRE1 and XBP-1s) in cortical neurons but not in thalamic neurons. This discrepancy may arise from region-specific factors: (1) cellular heterogeneity—the cortex, being metabolically active and critical for higher-order functions, is inherently more vulnerable to ER stress and neuroinflammation, while thalamic neurons, which relay sensory/motor signals, may exhibit enhanced stress tolerance through baseline ER chaperones or alternative survival pathways (e.g., PERK/ATF4) [71, 72]; (2) BBB permeability—the cortex experiences greater iRBC sequestration and BBB disruption in ECM, facilitating exosome delivery and amplifying cortical ER stress, whereas thalamic BBB integrity might limit exosome penetration [73]; (3) pathway divergence—the thalamus may prioritize non-IRE1 ER stress pathways (e.g., ATF6) to mitigate damage, as seen in other neurological disorders [74]. These regional differences highlight the complexity of ECM pathology and suggest that cortical neurons may serve as primary targets for exosome-mediated ER stress exacerbation. Future studies will assess exosome biodistribution, baseline ER stress markers, and non-IRE1 pathways to clarify these differences. These findings align with reports implicating CHOP-driven apoptosis in neurodegeneration [75–77], as *RE*/*AE* increased TUNEL^+^-NeuN^+^ and NeuN^+^-CHOP^+^ neurons in ECM mice, mirroring ER stress-induced hippocampal apoptosis via XBP-1/CHOP [78]. Mast cell-derived exosomes, akin to ceramide-mediated ER stress in eczema models [79], thus exacerbate neuronal injury in ECM through IRE1 pathway activation, highlighting cortical neurons as primary targets. Future studies should clarify exosome biodistribution and non-IRE1 pathway contributions to regional ER stress disparities.

In this study, *AE* treatment of *Pb*A-infected mice increased ECM incidence, exacerbated BBB disruption, neuronal injury, and neuroinflammation, and increased expression levels of ER stress-related factors (GRP78, CHOP, p-IRE1, and XBP-1) in cortical neurons compared to *RE* treatment. *AE* treatment also induced higher mRNA levels of GRP78, CHOP, and XBP-1 in iRBC-stimulated HT-22 neuronal cells. Prior studies suggest that exosomes can modulate ER stress-related pathways via microRNAs targeting the 3′ untranslated regions (UTRs) of key genes [80–82]. To explore this mechanism, we compared miRNA profiles between *AE* and *RE* using miRNA-seq, identifying 128 differentially expressed microRNAs. Among these, three miRNAs (miR-330-3p, miR-185-5p, and miR-379-5p)—downregulated in *AE* relative to *RE*—directly target and suppress GRP78 and XBP-1s [40–42]. Conversely, six miRNAs (miR-155-5p, miR-423-3p, miR-187-3p, miR-29c-3p, miR-188-5p, and miR-192-5p)—upregulated in *AE*—indirectly enhance GRP78 and CHOP expression [43–48]. These findings align with RNA-seq and qPCR data from resting versus degranulated bone marrow-derived mast cells (BMMCs), which identified 47 differentially expressed miRNAs (11 upregulated, 36 downregulated) [83]. Further, dysregulation of miR-29c-3p on cerebral mast cells contribute to the development and progression of meningiomas [84, 85]. It was reported that miR-29c-3p mediated renal epithelial cells injury by activating ER stress pathways [46], consistent with our miRNA-seq data showing reduced miR-29c-3p in *AE*. It was demonstrated that *P. falciparum* exosomes deliver miR-155-5p to human endothelial cells, amplifying inflammatory responses—a mechanism consistent with our observation that *AE*-enriched miR-155-5p exacerbates ER stress in neurons [86]. Although our study identifies mast cell-derived exosomal miRNAs (e.g., miR-29c-3p and miR-155-5p) as potential regulators of neuronal ER stress in ECM, functional validation (e.g., neuron-specific ER stress reporters, miRNA knockout models) is needed to establish causality. Technical limitations currently preclude mechanistic studies such as in vivo ER stress silencing or exosomal miRNA functional assays, highlighting critical directions for future research.

Pharmacological inhibition of macrophage-derived exosome release via GW4869 had been shown to mitigate silica-induced lung fibrosis by suppressing ER stress [87], while blockade of pericyte-derived exosomes with GW4869 improved mitochondrial function and attenuated neuronal injury in spinal cord injury models [88], collectively validating exosome targeting as a therapeutic strategy against ER stress-associated pathologies. In contrast, our findings demonstrated that mast cell-derived exosomes aggravate ECM-related neuronal injury through ER stress activation. To establish causality, future studies should implement exosome inhibition approaches (e.g., GW4869) as successfully demonstrated in fibrosis and neurotrauma research [87, 88]. Notably, miRNA-seq analysis revealed *AE*-enriched miRNAs (miR-155-5p, miR-29c-3p) previously implicated in ER stress amplification, further substantiating the mechanistic connection between exosomal miRNAs and CM neuronal injury progression. These insights underscore the therapeutic potential of modulating exosomal pathways in CM management. However, current experimental limitations prevent definitive in vivo validation of mast cell-derived exosome inhibition (e.g., via GW4869) to confirm their pathogenic role in ER stress-mediated neuronal injury.

This study has several limitations. First, experimental constraints necessitated the use of exosomes from P815 mast cells rather than BMMCs to assess their role in ECM-associated neuronal injury; future work should utilize BMMC-derived exosomes to validate their pathological contributions. Second, the mechanisms linking mast cell-derived exosomal miRNAs to ER stress targeting in ECM neurons remain undefined. Third, longitudinal profiling of ER stress dynamics (e.g., GRP78, p-IRE1, XBP-1, and CHOP at early, mid-, and late infection stages) is warranted. Fourth, while cortical—but not thalamic—IRE1-driven ER stress correlated with neuronal injury during ECM progression, the regional specificity of this phenomenon requires mechanistic clarification. Addressing these gaps will deepen insights into mast cell–neuron cross-talk in ECM pathogenesis. Our findings underscore the therapeutic promise of targeting mast cell-derived exosomes or ER stress pathways to alleviate ECM-related neuronal injury. Activated mast cells release exosomes enriched with ER stress-promoting miRNAs (e.g., miR-155-5p, miR-29c-3p, or miR-330-3p), identifying actionable intervention points. Pharmacological strategies—such as inhibiting exosome release/uptake (via mast cell stabilizers [cromolyn sodium, ketotifen] or GW4869) or suppressing ER stress (e.g., 4-PBA)—could synergize with antimalarial therapies to attenuate neuroinflammation and apoptosis. Furthermore, neutralizing pathogenic exosomal miRNAs or augmenting neuroprotective miRNAs may restore ER homeostasis, enabling precision therapeutic approaches. Integrating these strategies with current antimalarial regimens holds potential to improve survival and mitigate long-term neurological sequelae in severe malaria.

## Conclusions

In summary, this study highlights the role of mast cell-derived exosomes in exacerbating ECM-related neuronal injury. We demonstrated that exosomes from P815 mast cells—particularly activated exosomes (*AE*)—aggravated neuronal damage during ECM progression, mediated at least partially by ER stress activation. While the precise mechanisms by which mast cell-derived exosomes trigger neuronal ER stress require further investigation, our findings provide novel insights into mast cell–neuron interactions and their contribution to the pathogenesis of ECM neuronal injury.

## Supplementary Information

Supplementary Material 1: Supplementary Fig. 1. Heatmap analysis of differentially expressed microRNAs between resting and LPS-activated P815 mast cell-derived exosomes. miRNA-seq profiling identified 128 differentially expressed microRNAsacross biological replicates. Expression levels were visualized as *Z*-score-normalized counts, with gradient coloration from lowto highfluorescence intensity.

## Data Availability

Data supporting the main conclusions of this study are included in the manuscript.
